# Reconstitution of T cell‐mediated immunity by umbilical cord‐derived mesenchymal stem cells in ulcerative colitis

**DOI:** 10.1002/ctm2.70452

**Published:** 2025-08-21

**Authors:** Xiaoying Luo, Jieping Deng, Xiaoke Jiang, Jun Mi, Yangqiu Bai, Huimin Zhang, Yalong Li, Min Liu, Conghui Cai, Pengju Li, Huanrong Huang, Yueping Xu, Yiwen Qin, Yang Mi, Hui Ding, Zhiyu Yang, Yue Wu, Zhenjuan Li, Ling Lan, Lida Zhang, Li Wang, Guobing Chen, Han Yue, Oscar Junhong Luo, Bingyong Zhang

**Affiliations:** ^1^ Department of Gastroenterology Henan Provincial People's Hospital, People's Hospital of Zhengzhou University, Zhengzhou University Zhengzhou China; ^2^ Department of Microbiome Laboratory Henan Provincial People's Hospital, People's Hospital of Zhengzhou University, Zhengzhou University Zhengzhou China; ^3^ Department of Systems Biomedical Sciences School of Medicine Jinan University Guangzhou China; ^4^ Department of Stem Cell Research Center Henan Key Laboratory of Stem Cell Differentiation and Modification Henan Provincial People's Hospital, People's Hospital of Zhengzhou University, Zhengzhou University Zhengzhou China; ^5^ Department of Gastroenterology Luoyang Central Hospital Affiliated to Zhengzhou University Luoyang China; ^6^ Department of Gastroenterology Puyang People's Hospital Puyang China; ^7^ Department of Henan Key Laboratory for Helicobacter pylori and Digestive Tract Microecology Marshall Medical Research Center The Fifth Affiliated Hospital of Zhengzhou University Zhengzhou China; ^8^ Department of Gastroenterology Heart Center of Henan Provincial People's Hospital, Central China Fuwai Hospital of Zhengzhou University Zhengzhou China; ^9^ Department of Stem Cell and Regenerative Medicine Research and Translation Center Henan Academy of Innovations in Medical Sciences Zhengzhou China; ^10^ Department of Microbiology and Immunology Institute of Geriatric Immunology, School of Medicine, Jinan University Guangzhou China

**Keywords:** immune regulation, single‐cell RNA sequencing, T lymphocyte, ulcerative colitis, umbilical cord‐derived mesenchymal stem cell

## Abstract

**Background:**

Ulcerative colitis (UC) is an agnogenic chronic intestinal inflammatory disease. Umbilical cord‐derived mesenchymal stem cell (UMSC) is a potential therapeutic approach against UC; however, the mechanisms underlying their efficacy for UC remain unclear.

**Methods:**

We performed a single‐arm clinical trial with 6 months follow‐up to assess the efficacy of UMSC in patients with moderate to severe left‐sided UC. The 26 enrolled patients were administered two UMSC doses intravenously. Pre‐ and post‐therapy colon biopsy specimens were analysed by single‐cell RNA sequencing (scRNA‐seq). Dextran sulphate sodium (DSS)‐induced colitis mouse models with or without UMSC injection were used to delineate colon inflammation and T cell function.

**Results:**

In the clinical trial, the clinical response/remission rates were 80.8/46.2% and 75.0/37.5% after 2 and 6 months of therapy, respectively. Endoscopic and histological examinations showed improvement of colonic mucosa after UMSC therapy in responders. scRNA‐seq data showed that UMSC therapy may suppress pro‐inflammatory features of T lymphocytes and alleviate inflammatory responses by inhibiting the interaction of T cells with B and myeloid cells. In the murine experiment, UMSCs suppressed DUOX2‐mediated oxidative stress to attenuate DSS‐induced colitis by regulating T cell‐mediated immunity.

**Conclusion:**

UMSC therapy primarily modulates T cell‐mediated immunity to achieve gut mucosal immune reconstitution and maintain mucosal barrier integrity, thereby achieving effective UC recovery.

**Highlights:**

- UMSCs effectively induce clinical remission in patients with active UC via T cell‐mediated immune reconstitution.
- scRNA‐seq analyses further revealed that UMSC therapy suppressed pro‐inflammatory features of T cells and alleviated inflammatory responses by inhibiting the interaction of T cells with B and myeloid cells
- UMSCs suppressed DUOX2‐mediated oxidative stress to attenuate DSS‐induced colitis by regulating T cell‐mediated immunity.

## Graphical abstract

1. UMSCs effectively induces clinical remission in patients with active UC via T cell‐mediated immune reconstitution.

2. The scRNA‐seq analyses revealed that UMSC therapy suppressed pro‐inflammatory features of T cells and alleviated inflammatory responses by inhibiting interaction of T cells with B and myeloid cells.

3. UMSCs suppressed DUOX2‐mediated oxidative stress to attenuate DSS‐induced colitis by regulating T cell‐mediated immunity.

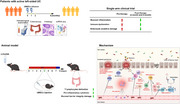

## 1 INTRODUCTION

Ulcerative colitis (UC) is a contiguous chronic inflammatory disease of colorectal mucosa, characterised by frequent relapse, dysfunction and anatomical changes in the colon and rectum, and related to an increased risk of colorectal cancer, resulting in impaired quality of life.^1^ UC is well known for its complex aetiology. According to mechanistic studies, UC is largely attributable to dysregulation of intestinal immune function and disturbance of the gut microbiota caused by genetic predisposition and environmental factors.^2^ Despite therapeutic advances, such as corticosteroids, immunosuppressive drugs, biological agents, small‐molecule therapies and faecal microbiota transplantation,^3^, ^4^ many refractory patients respond poorly to the available therapeutic options. Therefore, alternative and effective therapeutic strategies are urgently required.

Mesenchymal stem cells (MSCs), which can be derived from multiple tissues (e.g., adipose, bone marrow, dental pulp, umbilical cord) of the body, are a potential treatment for various diseases, including autoimmune diseases, owing to their high degree of self‐renewal, multidirectional differentiation potential and modulation of immune homeostasis and microbial dysbiosis.^5^, ^6^, ^7^ Animal experimental research suggests that MSCs improve colonic mucosa inflammation.^8^, ^9^ Indeed, adipose‐derived MSCs (AD‐MSCs) effectively induce clinical remission among patients with Crohn's disease (CD) and perianal fistulas.^10^ Local injection of bone marrow‐derived MSCs (BM‐MSCs) effectively mediated clinical and radiographic healing among patients with perianal fistulising CD and improved endoscopic performance in patients with UC.^11^, ^12^ Compared with other MSCs (including AD‐MSCs and BM‐MSCs), umbilical cord‐MSCs (UMSCs) may be a more suitable therapeutic approach for inflammatory bowel disease (IBD), owing to the ease of extraction and culture, reduced ethical concerns, as well as lower expression of human leukocyte antigen‐II and major histocompatibility complex (MHC) class I molecules in UMSCs.^13^ However, limited clinical trials have investigated UMSC therapy against patients with active UC; moreover, the underlying mechanism remains poorly understood.

To assess the efficacy of UMSC therapy for UC and investigate how UMSCs protect the intestinal mucosa barrier by modulating immune cells, especially T lymphocytes, we conducted a clinical trial, followed up with 6 months of observation and single‐cell transcriptome analysis of the sigmoid mucosal tissues of patients pre‐ and post‐UMSC therapy. Additionally, we constructed a dextran sulphate sodium (DSS)‐induced colitis mouse model and then administered UMSCs to validate the key mechanistic factors. Our data illustrate that UMSCs effectively induce clinical remission among patients with moderate to severe UC by reconstituting T lymphocyte‐mediated immunity to restore epithelial cell differentiation and restrict colonic inflammation. Our research provides clinical evidence for the efficacy and mechanisms of UMSCs as a potential therapeutic approach against UC.

## 2 METHODS

### 2.1 Clinical trial design

This single‐centre, nonrandomised, non‐placebo‐controlled, prospective before‐after clinical study was conducted at Henan Provincial People's Hospital between March 2018 and March 2023. The study was registered at the Chinese Clinical Trial Registry (www.chictr.org.cn, ChiCTR1900026035) and approved by the Committee on the Ethics of Henan Provincial People's Hospital (NO.: (2018) NO. 03‐01). The inclusion criteria for patients with moderate to severe left‐sided UC were as follows: (1) age 18–65 years; (2) active left‐sided UC diagnosed in accordance with the consensus opinions on the diagnosis and treatment of IBD^14^; (3) agreement to participate in the whole experimental period. The criteria for excluding participants were as follows: (1) mild, initial‐ or explosive‐onset UC; (2) allergic constitution, or allergies to the components of the drug treatment; (3) chronic colitis caused by other causes such as radiation‐induced colitis, drug‐induced colitis, unexplained colitis and CD; (4) pregnancy or lactation period; (5) a history of mental illness, malignant tumours or haematological diseases, serious cardiovascular or cerebrovascular system diseases; (6) a history of acute cerebrovascular disease or myocardial infarction in the past 3 months, uncontrollable hypertension (>180/110 mmHg), severe cardiac insufficiency based on the New York Heart Function Classification IV or ejection fraction <30%, and malignant arrhythmia; (7) comorbidity with other autoimmune diseases; (8) liver and kidney dysfunction; (9) participation in any other clinical trial within 3 months. Accordingly, 26 patients with UC were enrolled. Prior to carrying out the study, we obtained written informed consent from all individuals. Clinical information, including demographics, disease severity, disease duration, previous medications and endoscopic data, was recorded on the day of the initial admission.

### 2.2 Human UMSC therapy and procedures

Recent case reports and clinical trials have shown that the safe dose of UMSC intravenous infusion ranges from approximately 0.5 × 10^6^ to 10^8^ cells for various diseases, such as CD,^15^ liver cirrhosis,^16^ aging frailty,^17^ heart failure,^18^ osteoarthritis^19^ and endometriosis.^20^ Moreover, our previous clinical trial confirmed a safe and effective dosage (1 × 10^6^ cells/kg, once a month, 2 consecutive months) for active UC.^21^ Therefore, in this study, patients with UC were administrated with weight‐based doses of human UMSCs (1 × 10⁶ cells/kg). The therapeutic procedure was as follows: patients were treated with an intravenous infusion of UMSCs dissolved in 100 mL of normal saline under electrocardiographic monitoring, with one dose administered per month for 2 consecutive months. Following UMSC administration, patients received a full dose of 5‐aminosalicylic acid (4 g/day, orally).

### 2.3 Outcomes

The enrolled patients were followed up for 2 and 6 months. Clinical outcomes were recorded at baseline, 2 months and 6 months. Mayo and UC endoscopic index of severity (UCEIS) scores were calculated for all patients according to the previously described methods.^22^, ^23^ The Nancy histological index from colon biopsy specimens was used to assess histological disease activity in patients with UC.^24^ Patients exhibiting a clinical response were defined as those with a reduction in their total Mayo score of ≥30% and of ≥3 points compared with those at the baseline, a reduction in the rectal bleeding component of Mayo score of at least one point, or a rectal bleeding subscore of 0 or 1. Clinical remission was defined as a total Mayo score ≤2 and an endoscopy subscore ≤1.^23^

### 2.4 scRNA‐seq data processing, clustering and cell‐type annotation

Based on the 75% clinical response rate at 6 months post‐therapy in this clinical trial, of the 26 patients with moderate to severe left‐sided UC, we collected a total of 22 colonic mucosal samples for scRNA‐seq from the eight patients, six of which were responders and 2 of which were non‐responders at 6 months post‐therapy. More details are provided in the Supplementary Methods.

Raw FASTQ files were processed using the BD Rhapsody WTA Analysis Pipeline (Doc ID: 47383 Rev. 9.0). Count matrices were imported into the Seurat R package (v4.3.0) for downstream analysis and visualisation.^25^ Only cells with >200 and <6000 detected genes, of which <25% were mitochondrial genes, were retained for analysis (Figure S1B). All processed data were consolidated into one data object. Genes were normalised and scaled, and samples were integrated using the Harmony algorithm^26^ for further analysis. The dataset included normalised expressions of 32 250 genes from 236 239 single cells. Principal component analysis was performed, and the top 20 components were used for clustering (using the Find Clusters function with a resolution of 0.6). Seven primary cell types were identified. The top 10 cluster‐specific genes were extracted using the Find All Markers function for cell type annotation. Each main cell type was then isolated for more detailed annotation (Table S2). The raw sequence data reported in this paper have been deposited in the Genome Sequence Archive (https://ngdc.cncb.ac.cn/gsa/; GSA‐Human: HRA006099).

### 2.5 Animal experiment design

Male C57 mice (*n* = 27; 6–8 weeks old; 18–22 g) were obtained from SiPeiFu Biotechnology Co., Ltd. (Beijing, China). Animal experiments were approved by the Committee on the Ethics of Animal Experiments of Zhengzhou University (NO.: ZZU‐LAC20220422[16]). Mice were randomly assigned to three groups (*n* = 9 per group): vehicle, DSS and DSS+UMSCs. According to a previous study,^27^ mice in the DSS and DSS+UMSCs groups were given autoclaved drinking water containing 2.5% DSS for 7 days, followed by autoclaved water alone for 1 day. The DSS+UMSCs group was then additionally treated with UMSCs (1 × 10^6^ cells per mouse) via caudal vein injection on Day 4. On Day 8, mice in all three groups were euthanised, and the colon and spleen tissues were harvested.

### 2.6 Statistical analysis

Normally distributed continuous variables were presented as mean ± standard deviation and compared using paired‐sample *t*‐tests. Non‐normally distributed continuous variables were presented as median (interquartile range), and categorical variables as numbers and proportions (%) and were compared using the Wilcoxon (Mann–Whitney) test. SPSS software 21.0 was performed for all statistical analyses. A two‐tailed *p* value < 0.05 was considered statistically significant. Additional details are provided in the Supplementary Material.

## 3 RESULTS

### 3.1 UMSC therapy effectively induces remission in UC

**FIGURE 1:**
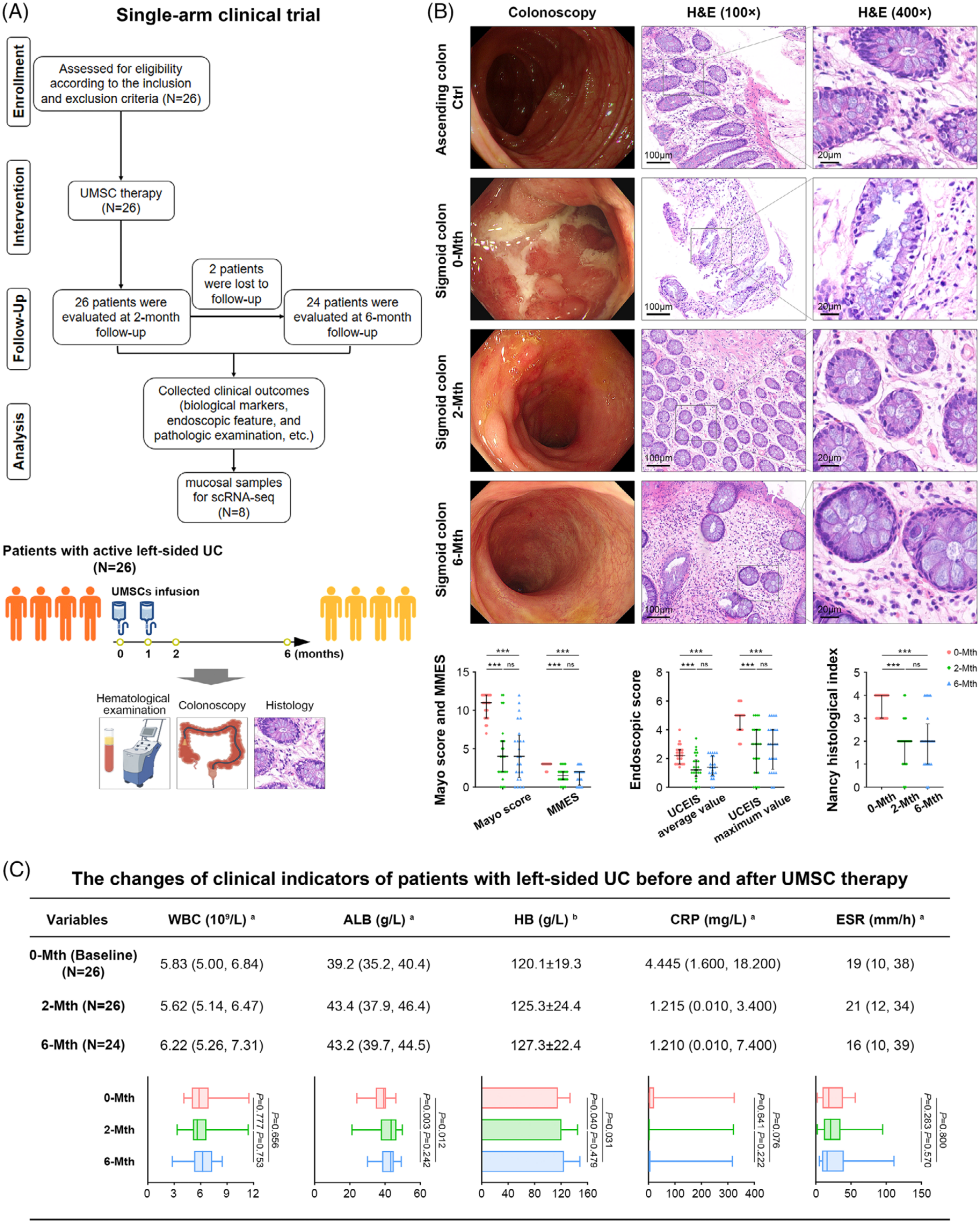
UMSC therapy effectively remits UC. All 26 patients with moderate to severe left‐sided UC received UMSC infusions twice (once per month) and were evaluated over 2‐ and 6‐month follow‐up periods. (A) The flowchart of the single‐arm clinical trial for patients with moderate to severe left‐sided UC. (B) Representative high definition colonoscopic images and H&E staining for the colon biopsies (100×, scale bar: 100 µm; 400×, scale bar: 20 µm) in the ascending colon and sigmoid colon of patients at pre‐therapy (*n* = 26), 2 months post‐therapy (*n* = 26) and 6 months post‐therapy (*n* = 24). The Mayo score, modified Mayo endoscopic score (MMES), ulcerative colitis endoscopic index of severity (UCEIS) and Nancy histological index were quantified in the graphs (below). A paired non‐parametric test was used to compare the difference between the three groups (0‐Mth, 2‐Mth and 6‐Mth). ^*^ *p *< 0.05, ^**^ *p *< 0.01 and ^***^ *p *< 0.001 versus the 0‐Mth group. (C) Changes in clinical indicators in patients with UC, including white blood cell (WBC), albumin (ALB), haemoglobin (HB), C‐reactive protein (CRP) and erythrocyte sedimentation rate (ESR) at pre‐therapy (*n* = 26), 2‐month post‐therapy (*n* = 26) and 6‐month post‐therapy (*n* = 24) were quantified (below). Data are the median (interquartile range) or mean ± standard deviation (SD). ^a^Paired non‐parametric test and ^b^paired‐samples *t*‐test were used to compare the difference between the three groups (0‐Mth, 2‐Mth and 6‐Mth).

All 26 patients with moderate to severe left‐sided UC, including 16 males and 10 females, received two doses (1 × 10^6^ cells/kg body weight per month) of UMSC infusions and were evaluated at 2‐ and 6‐month follow‐ups. Twenty‐four of the 26 patients underwent a 6‐month follow‐up period, whereas two patients only participated in the 2‐month follow‐up period (Figure 1A). The mean age, body mass index and disease duration among patients were 53.5 years, 22.1 kg/m^2^ and 5.0 years, respectively. Of these, 17 patients were diagnosed with moderately active UC and nine patients were diagnosed with severely active UC at baseline. All patients regularly took a full dose of 5‐aminosalicylic acid (4 g/day, orally); eight (30.8%) had previously been treated with glucocorticoids, four (15.4%) had previously used glucocorticoids and immunosuppressants, and one (3.8%) had previously received biological agents. All enrolled patients were dissatisfied with or intolerant of previous treatments. The baseline characteristics of the enrolled patients were presented in Table S1.

During the observation period, 26 patients received two UMSC infusions. The proportions of clinical response and remission were 80.8% (21 out of 26) and 46.2% (12 out of 26) after 2 months of therapy and 75.0% (18 out of 24) and 37.5% (nine out of 24) after 6 months of therapy. Endoscopic features and histological examination showed that the inflamed colonic mucosa of patients with active UC improved at 2 and 6 months after therapy in the responders. The Mayo score, modified Mayo endoscopic score (MMES), average and maximum values of the UCEIS and Nancy histological index for UC all decreased at 2‐ and 6‐month after therapy in comparison with those at baseline (Figure 1B). According to paired‐samples *t*‐test and paired non‐parametric test analysis, UMSC therapy significantly improved albumin (ALB) and haemoglobin levels in patients (Figure 1C).

**FIGURE 2:**
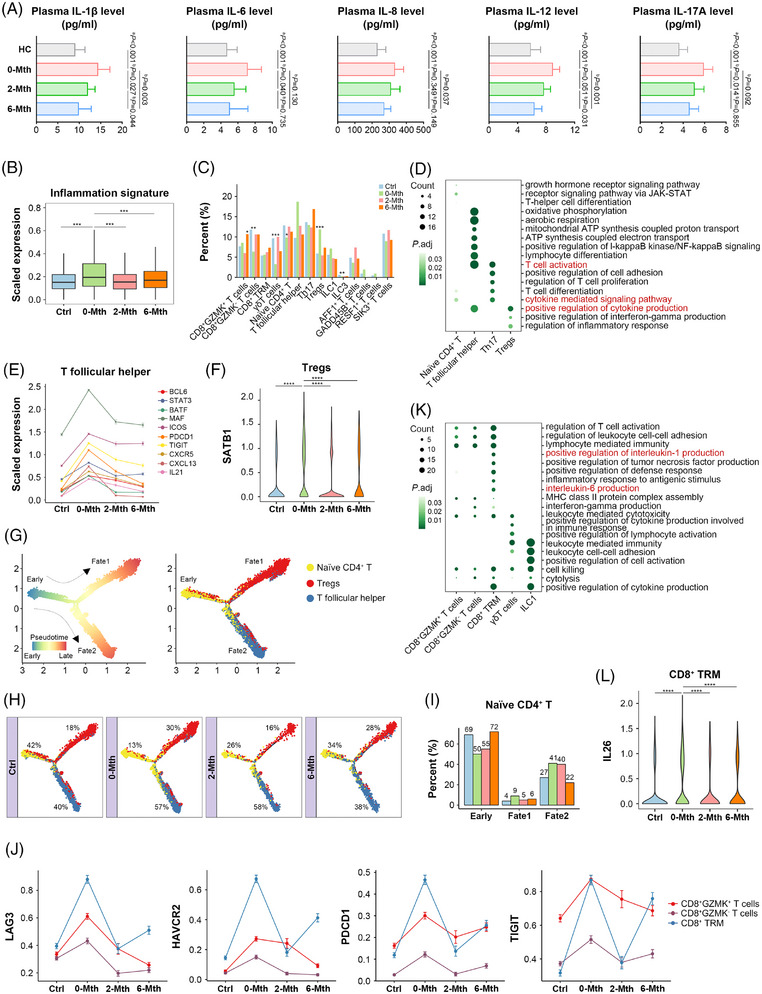
UMSC therapy inhibits T lymphocyte pro‐inflammatory features and reverses CD8^+^ T cell functional exhaustion. (A) ELISA detection of plasma inflammatory cytokines (including IL‐1β, IL‐6, IL‐8, IL‐12 and IL‐17A) in patients with UC at pre‐therapy (*n* = 26), 2 months post‐therapy (*n* = 26) and 6 months post‐therapy (*n* = 24) and compared with those in healthy individuals (*n* = 26). ^a^Student's *t*‐test and ^b^Paired‐samples *t*‐test were used to compare differences between healthy control (HC) and 0‐Mth groups and between the three groups (0‐Mth, 2‐Mth and 6‐Mth). (B) Boxplots of inflammation signature scores of cells within non‐inflamed (Ctrl, *n* = 8) and inflamed (0‐Mth, *n* = 8) mucosal biopsy specimens in patients with UC pre‐therapy, as well as previously inflamed sites 2 (2‐Mth, *n* = 2) and 6 (6‐Mth, *n* = 4) months after therapy. (C) Bar plots of T lymphocyte subset proportions among Ctrl, 0‐Mth, 2‐Mth and 6‐Mth groups. See Table S3. (D) Selected GO terms with significant enrichment for shared up‐regulated genes in naïve CD4^+^ T, T follicular helper (Tfh), T helper (Th) 17 and Tregs subsets. See Table S4. (E) Average normalised expression of activation‐related genes expressed with Tfh cells; (F) violin plot of normalised expression of SATB1 in Tregs; and (G and H) differentiation trajectories of naïve CD4^+^ T, Tfh and Tregs clusters across Ctrl, 0‐Mth, 2‐Mth and 6‐Mth groups. Trajectories of g split by groups. Percentages indicate the proportion of cells mapped to each branch of the trajectory. (I) Percentage of naïve CD4^+^ T cells mapped to each branch of the trajectory across the four groups. (J) Average normalised expression levels of exhaustion and cytotoxicity‐related genes within three CD8^+^ T subsets across the four groups. (K) Selected GO terms with significant enrichment for the shared up‐regulated genes in CD8^+^GZMK^+^ T cells, CD8^+^GZMK^−^ T cells, CD8^+^ TRM, γδT cells and ILC1. See Table S4. (L) Violin plot of normalised expression of IL‐26 in CD8^+^ TRM cells across the four groups. Asterisks in B, C and L indicate significant differences in pairwise comparisons (Wilcoxon test; ^*^ *p *< 0.05, ^**^ *p *< 0.01 and ^***^ *p *< 0.001; boxplots: 25, 50 and 75% quantiles; error bars: SD). In D and K, differential gene expression was compared between 0‐Mth and Ctrl groups, as well as between 0‐Mth and combined 2‐Mth+6‐Mth groups. GO enrichment analysis and the functional enrichment plot focused on the common up‐regulated genes.

Additionally, 26 healthy individuals were recruited from the healthy control group and matched by age and sex. Compared with the healthy control group, the levels of plasma interleukin (IL)‐1β, IL‐6, IL‐8, IL‐12 and IL‐17A were increased in patients with UC. These levels then decreased 2 and 6 months post‐therapy, suggesting that UMSC therapy may effectively inhibit the production of inflammatory cytokines in patients with UC (Figure 2A).

### 3.2 Single‐cell analysis of colonic mucosa in patients with UC following UMSC therapy

To understand the cellular and molecular mechanisms underlying UMSC therapy for UC, we collected 22 colonic mucosal samples for scRNA‐seq from eight patients, six of whom were responders and two of whom were non‐responders according to the clinical response rate (75.0%) at 6 months post‐therapy. Mucosal biopsies were collected from clinically non‐inflamed (Ctrl; *n* = 8) and inflamed (0‐Mth; *n* = 8) sites prior to therapy, and mucosal biopsies were harvested from previously inflamed sites 2 (2‐Mth; *n* = 2) and 6 (6‐Mth; *n* = 4) months after treatment for comparison (Figure S1A–C). After quality control, 236 239 high‐quality single cells were retained for further analysis (Figure S1D). Based on canonical marker genes, the cells were initially categorised into seven major cellular compartments (Figure S1E–G and Table S2). Compared with the control group, the expression of inflammation‐associated genes was significantly increased in the inflamed mucosa of the 0‐Mth group but subsequently reduced in both 2‐Mth and 6‐Mth groups, which was consistent with the changes in endoscopic scores (Figure 2B). We further dissected the top five major cell compartments (excluding mast and glial cells owing to their low abundance), resulting in 52 cell subtypes, including 12 epithelial, 12 stromal, 14 T lymphocyte, eight B lymphocyte and nine myeloid cell subtypes (Figure S1H).

### 3.3 UMSC therapy inhibits the pro‐inflammatory features of T cells and reverses functional exhaustion of CD8^+^ T cells

Although the aetiology of UC remains unknown, immunological derangement may play a crucial role in the progression of UC. Therefore, we focused on the characteristics of T lymphocytes and their effects on B lymphocytes, myeloid cells and epithelial cells after UMSC therapy. Fourteen T lymphocyte subsets were identified and annotated (Figures S1H and S2A). UMSC therapy exerted distinct effects on different T lymphocyte subsets. Compared with the control group, T follicular helper (Tfh) cells and regulatory T cells (Tregs) were more prevalent in the 0‐Mth group, whereas naïve CD4^+^ T cells were less abundant; these effects were reversed following UMSC therapy (Figure 2C). Gene ontology (GO) analysis showed that the pathways associated with T cell activation and cytokine production in T helper (Th) 17 cells, Tfh cells and Tregs were inhibited after UMSC therapy (Figures 2D and S2B and Table S2).

IL‐17 is a pro‐inflammatory cytokine primarily secreted by Th17 cells that persistently triggers a T cell‐induced inflammatory response when unrestrained.^28^ JAK3, TYK2, STAT3 and IL17A were highly expressed in Th17 cells in the 0‐Mth group but reduced in 2‐Mth and 6‐Mth groups, indicating that UMSC therapy may reduce IL‐17 and inhibit the JAK–STAT3 pathway to attenuate colonic inflammation (Figure S2C).

In addition, the expression levels of Tfh cell development‐ and differentiation‐related genes (e.g., BCL6, STAT3, MAF and ICOS) and chemotaxis‐related genes (e.g., CXCR5 and CXCL13) were up‐regulated in the 0‐Mth group and down‐regulated in 2‐Mth and 6‐Mth groups, suggesting that UMSC therapy inhibited the development and differentiation of Tfh cells and Tfh cell migration to the germinal centre (GC) (Figures 2E and S2B,D). We also observed high expression of IL‐21 and SATB1 in Tfh cells and Tregs, respectively, which may result in functional impairment of Tregs.^29^, ^30^, ^31^ These effects were reversed by UMSC therapy, indicating that UMSC therapy may help restore Treg function and modulate the mucosal immune response (Figure 2E,F). Furthermore, cell trajectory analysis revealed that naïve CD4^+^ T cells were mapped to the early pseudotime, whereas Tregs and Tfh cells progressed towards Fate1 and Fate2, respectively (Figure 2G). The population of naïve CD4^+^ T cells in the early state decreased, with an increased proportion differentiating towards Tregs and Tfh cells, particularly towards Tfh cells. However, the proportions of naïve CD4^+^ T cells in the early state gradually increased in both 2‐Mth and 6‐Mth groups (Figure 2H,I). These data indicate that UMSC therapy may inhibit the differentiation of naïve CD4^+^ T cells into Tregs and Tfh cells while potentially suppressing Tfh cell activation and restoring Treg function.

Notably, CD8^+^ T cells exhibited high expression of exhaustion‐related genes (e.g., LAG3, HAVCR2, PDCD1 and TIGIT), especially CD8^+^ TRM in the 0‐Mth group; however, the expression levels of these genes significantly declined after therapy (Figures 2J and S2E). Functional enrichment analysis revealed suppression of the signalling pathways governing the production of IL‐1 and IL‐6 in CD8^+^ TRM (Figures 2K and S2B). These results suggest that UMSC therapy inhibited the exhaustion of CD8^+^ TRM and the production of IL‐1 and IL‐6 to protect the immunological equilibrium. Moreover, a significant augment of IL‐26 was displayed in the CD8^+^ TRM of inflamed mucosa, which was reduced after therapy (Figure 2L). However, the precise role of IL‐26 in IBD remains unclear. Further analysis showed that IL10RB/IL20RA, the receptors for IL‐26,^32^ were mainly co‐expressed in DUOX2^+^CEACAM6^+^ enterocytes and transit amplifying (TA) cells, and IL‐26 appeared to induce CXCL8 expression within these cell subsets in epithelial cells (Figure S2F–H). These results suggest UMSC therapy suppresses the secretion of IL26 in CD8^+^ TRM to attenuate epithelium inflammation mediated by the IL10RB/IL20RA–CXCL8 axis.

### 3.4 UMSC therapy suppresses B lymphocyte migration and activation by inhibiting the Tfh cell‐mediated CXCR5–CXCL13 axis

**FIGURE 3:**
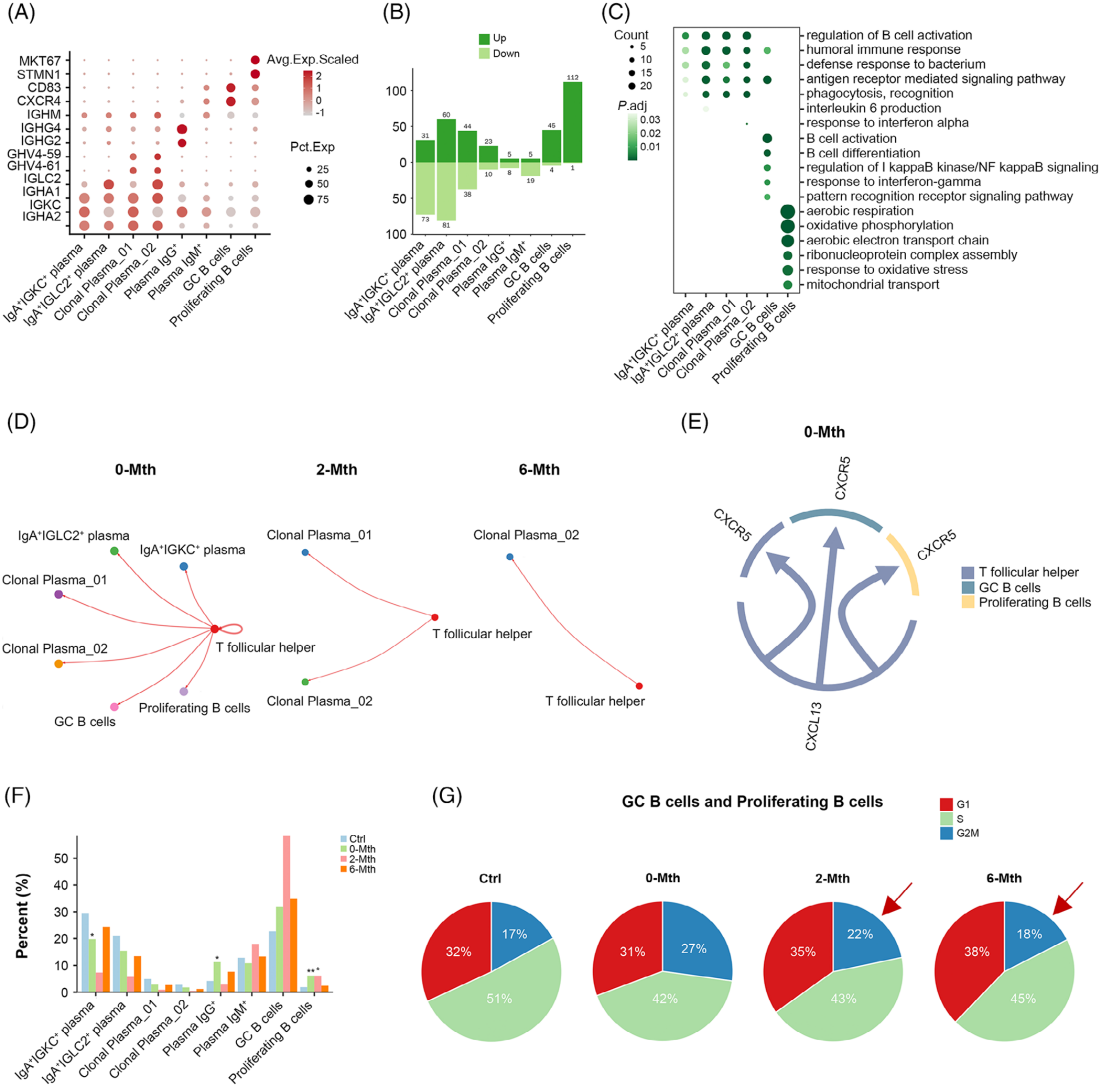
UMSC therapy suppresses B lymphocyte migration and activation by inhibiting the Tfh cell‐mediated CXCR5–CXCL13 axis. (A) Dot plot of marker genes for cell subtypes within B lymphocytes. (B) Bar plot illustrating the counts of up‐regulated and down‐regulated genes within B lymphocytes that were altered after UMSC therapy. (C) Selected GO terms with significant enrichment for shared up‐regulated genes in B lymphocyte subtypes. See Table S4. (D) Cell interaction network between Tfh and B cells within three groups (0‐Mth, 2‐Mth and 6‐Mth). (E) Chord diagram depicting interaction of the CXCL13–CXCR5 axis between Tfh and B cells in the 0‐Mth group. Edge colour corresponds to secretory cells, and the arrows point to receiver cells. (F) Bar plots of B lymphocyte subset proportions among the four groups (Ctrl, 0‐Mth, 2‐Mth and 6‐Mth). See Table S3. Asterisks indicate significant differences in pairwise comparisons (Wilcoxon test; ^*^ *p *< 0.05, ^**^ *p *< 0.01 and ^***^ *p *< 0.001). (G) Pie chart depicting the proportion of GC_B and proliferating B cells assigned to the three cell‐cycle phases.

Tfh cells support the differentiation and activation of B lymphocytes, and their interaction regulates the humoral immune response.^33^ Therefore, we investigated the characteristics of B lymphocytes and their interactions with Tfh cells after UMSC therapy. We classified eight subpopulations of B lymphocytes (Figure 3A) and identified genes that changed before and after therapy (Figure 3B). In the inflamed mucosa of patients with UC, most plasma and B lymphocyte subsets showed an activated and pro‐inflammatory state (e.g., regulation of B cell activation and regulation of IκB kinase/NFκB signalling) (Figure 3C). However, after UMSC therapy, the expression of genes related to these functions decreased in these cell populations, suggesting that UMSC treatment may help mitigate the activated and pro‐inflammatory state in B cell subpopulations. Considering the activation of Tfh cells in the 0‐Mth group, we conducted cell interaction analysis between Tfh cells and B lymphocytes. The results showed that Tfh cells in the 0‐Mth group interacted with GC_B and proliferating B cells, and these interactions were not observed post‐therapy (2‐Mth and 6‐Mth) (Figure 3D,E). Our data indicate that UMSC therapy exerts inhibitory effects on the CXCR5–CXCL13 axis, thereby reducing the infiltration of B lymphocytes.

Additionally, we analysed plasma cells under each clinical condition. High concentrations of IgG infiltration are typically observed in patients with UC.^34^ As expected, IgG^+^ plasma cells were significantly more abundant in the 0‐Mth group. However, after UMSC therapy, the proportion of IgG^+^ plasma cells decreased significantly, whereas that of GC_B cells increased significantly (Figure 3F). This indicates that UMSC therapy may influence the differentiation of GC_B cells. Furthermore, we analysed the cell cycles of GC_B and proliferating B cells. Compared with the 0‐Mth group, a greater number of GC_B and proliferating B subsets in the 2‐Mth and 6‐Mth groups were noted in the G1 phase of the cell cycle (Figure 3G). This suggests that GC_B and proliferating B subsets in the 0‐Mth group may have experienced cell cycle arrest, leading to an increase in the proportion of B cells in 2‐Mth and 6‐Mth phases (Figure 3F). These observations indicate that UMSC therapy may reduce the proportion of IgG^+^ plasma cells by inhibiting the differentiation and cell‐cycle progression of GC_B cells, thereby improving immune dysregulation in patients with UC.

### 3.5 UMSC therapy alleviates inflammatory responses by inhibiting the interactions between T Cells and myeloid cells

T cells play a crucial part in regulating the immune system after UMSC therapy; however, the mutual effects of T lymphocytes and myeloid cells have not been fully elucidated.

**FIGURE 4:**
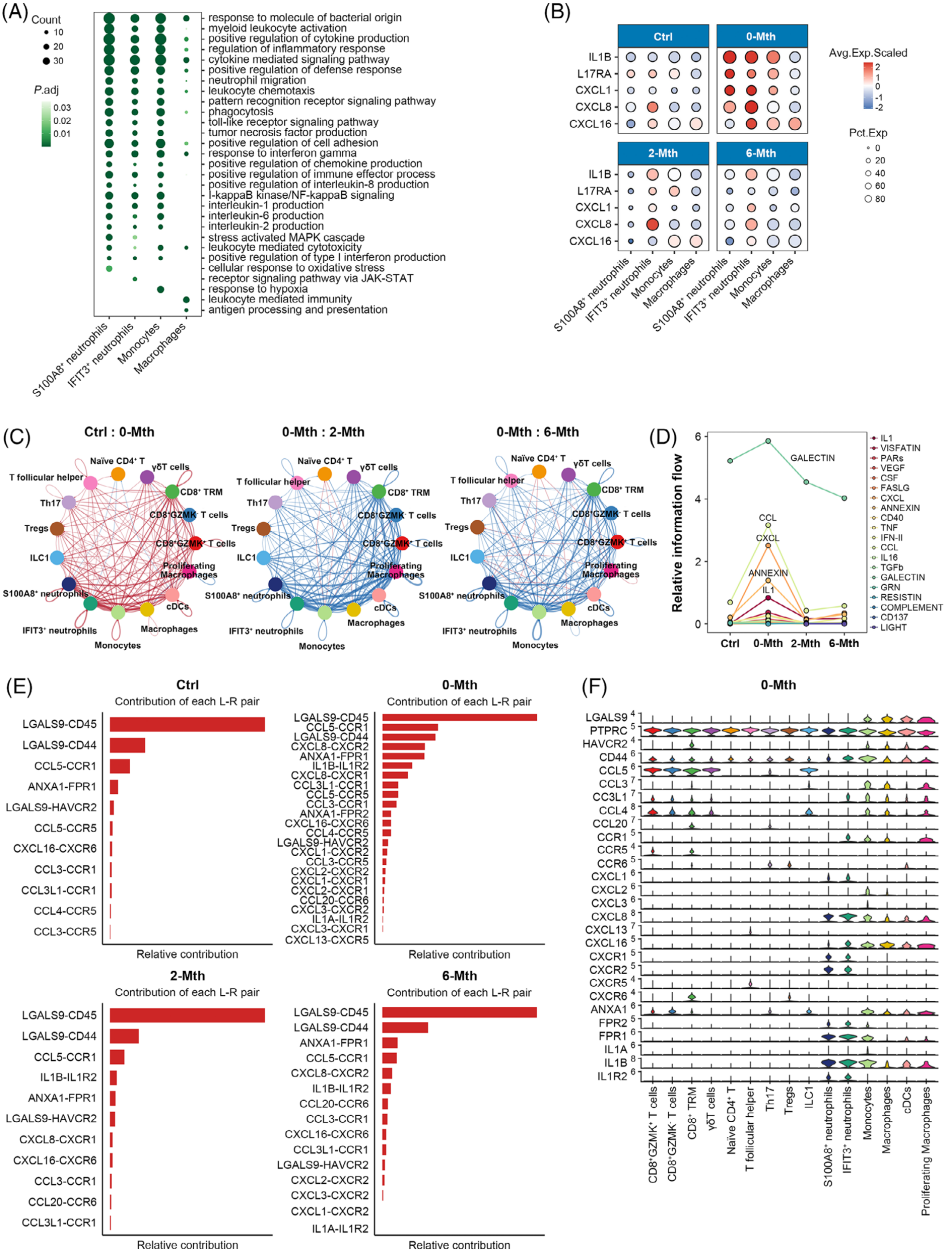
UMSC therapy alleviates inflammatory responses by inhibiting the interactions between T lymphocytes and myeloid cells. (A) Selected GO terms with significant enrichment for shared up‐regulated genes in myeloid subtypes. See Table S4. (B) Dot plot of average scaled expression of inflammation‐associated genes expressed within neutrophils, monocytes and macrophages across the four groups (Ctrl, 0‐Mth, 2‐Mth and 6‐Mth). (C) The cell interaction network between T lymphocytes and myeloid cells; (D) relative information flow of activation‐related signalling pathways; and (E) key ligand–receptor pairs within different myeloid subsets across the four groups (Ctrl, 0‐Mth, 2‐Mth and 6‐Mth). (F) Violin plot of normalised expression of activation‐related genes in the key ligand–receptor pairs within T lymphocytes and myeloid cells in the 0‐Mth group.

We characterised the effects of T lymphocytes on myeloid cells after UMSC therapy. We identified six myeloid cell subsets (Figure S3A). Among the myeloid cells, S100A8^+^ neutrophils were more abundant, but the proportions of macrophages and classical dendritic cells decreased in the 0‐Mth group; UMSC therapy reversed these effects (Figure S3B). For each myeloid cell subtype, we identified genes with significantly perturbed expression in UC but reverted to normal status after UMSC treatment for GO enrichment analysis (Figures 4A and S3C). Genes that were primarily related to cell activation and adhesion (e.g. myeloid leukocyte activation and positive regulation of cell adhesion) and inflammation regulation (e.g., regulation of the inflammatory response and positive regulation of chemokine production) were significantly suppressed by UMSC therapy in S100A8^+^ neutrophils, IFIT3+ neutrophils and monocytes subsets (Figure S3C).

To further explore inflammation‐related genes, we observed that the expression levels of inflammatory mediators and chemokines (e.g., IL‐1B, CXCL1 and CXCL8) were significantly decreased in both 2‐Mth and 6‐Mth groups (Figure 4B). These findings suggest that UMSC therapy may alleviate cellular damage and inflammatory responses by reducing the levels of these crucial inflammatory mediators. Next, we conducted interaction analysis between T cells and myeloid cells and found that interactions between cell subsets were generally suppressed after treatment, with this suppression persisting for up to 6 months post‐therapy (Figure 4C). The most significantly inhibited signalling pathways were GALECTIN, CCL, CXCL, ANNEXIN and IL1 (Figure 4D). Key ligand–receptor pairs that were suppressed included CCL5–CCR1, CXCL8–CXCR1/CXCR2, CCL3L1–CCR1 and IL1A/IL1B–IL1R2, which were particularly evident in S100A8^+^ neutrophils, IFIT3^+^ neutrophils and monocyte subsets (Figures 4E,F and S3D). Additionally, interactions between Tregs and myeloid cells in the 0‐Mth group were regulated by the CXCR6–CXCL16 axis. However, the expression of CXCR6 in Tregs significantly decreased at 2 and 6 months post‐treatment, indicating significant suppression of the CXCR6–CXCL16 axis. These findings suggest that UMSC therapy not only inhibits the expression of pro‐inflammatory factors and chemokines in myeloid cells but also reduces the infiltration of neutrophils and monocytes by suppressing the signalling pathways between T cells and myeloid cells, thereby modulating the inflammatory response and restoring immune homeostasis.

### 3.6 UMSC therapy improves enterocyte differentiation by reducing DUOX2‐mediated oxidative stress

**FIGURE 5:**
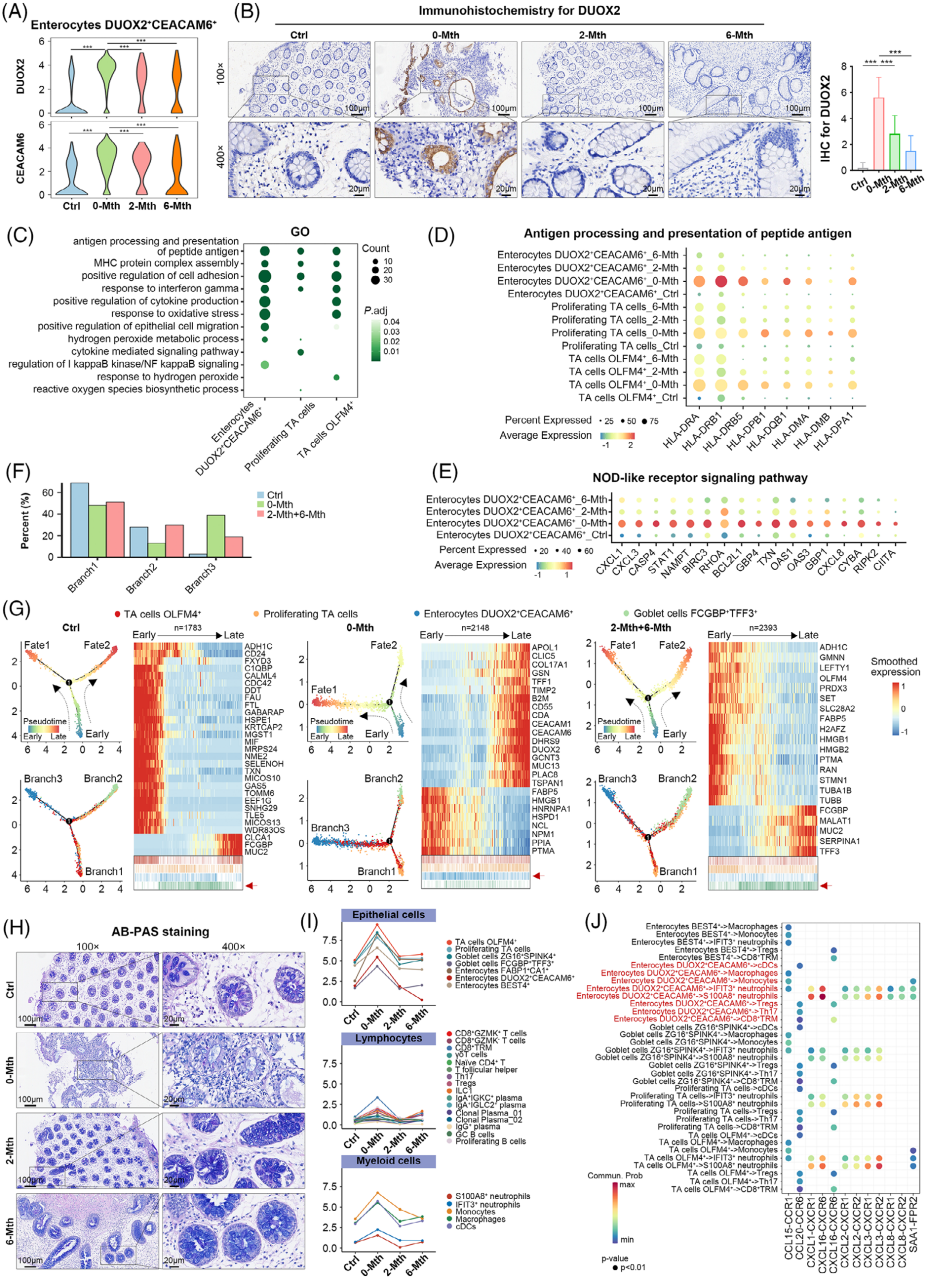
UMSC therapy improves enterocyte differentiation and mucosal barrier protection through immune reconstruction and reduced DUOX2‐mediated oxidative stress. (A) Violin plots of normalised expression for DUOX2 and CEACAM6 (Ctrl, *n* = 8; 0‐Mth, *n* = 8; 2‐Mth, *n* = 2; 6‐Mth, *n* = 6). Asterisks indicate significant differences in pairwise comparisons (Wilcoxon test; ^*^ *p *< 0.05, ^**^ *p *< 0.01 and ^***^ *p *< 0.001). (B) Representative immunohistochemistry (IHC) images for DUOX2 in colonic biopsy specimens (Ctrl, *n* = 26; 0‐Mth, *n* = 26; 2‐Mth, *n* = 26; 6‐Mth, *n* = 24) (100×, scale bar: 100 µm; 400×, scale bar: 20 µm). Semi‐quantified analysis of IHC‐stained area density for DUOX2 (right). Paired‐samples *t*‐test was used to compare differences between the four groups (^*^ *p *< 0.05, ^**^ *p *< 0.01 and ^***^ *p *< 0.001). The healthy control group is shown in Figure S4D (upper panels). (C) Selected GO terms that show significant enrichment for up‐regulated genes for DUOX2^+^CEACAM6^+^ enterocytes, proliferating TA cells and OLFM4^+^ TA cells in the 0‐Mth group. See Table S4. Dot plot illustrating the normalised expression of (D) genes enriched in the GO terms of antigen processing and presentation of peptide antigen within DUOX2^+^CEACAM6^+^ enterocytes, proliferating TA cells and OLFM4^+^ TA cells and (E) genes enriched in NOD‐like receptor signalling pathway in DUOX2^+^CEACAM6^+^ enterocytes across Ctrl, 0‐Mth, 2‐Mth and 6‐Mth groups. (F) Proportion of two TA subsets in each branch of the differentiation trajectory. (G) Cell differentiation trajectories for OLFM4^+^ TA, proliferating TA, DUOX2^+^CEACAM6^+^ enterocytes and FCGBP^+^TFF3^+^ goblet cells within Ctrl, 0‐Mth and combined 2‐Mth+6‐Mth groups, respectively. Heatmaps showed the scaled expression levels of genes with correlated and anti‐correlated expression profiles to the pseudotime; single cells are aligned according to early to late pseudotime mapping, and cell‐type annotations were shown below. (H) Representative images of AB‐PAS staining for goblet cells across the four groups (Ctrl, *n* = 26; 0‐Mth, *n* = 26; 2‐Mth, *n* = 26; 6‐Mth, *n* = 24) (100×, scale bar: 100 µm; 400×, scale bar: 20 µm); the healthy control group is shown in Figure S4D (lower panels). (I) Line graphs depicting changes in the average number of interactions within epithelial, lymphocyte and myeloid subtypes across Ctrl, 0‐Mth, 2‐Mth and 6‐Mth groups. (J) Bubble plot of intercellular communication within epithelial, T lymphocyte and myeloid cells in the 0‐Mth group. CXCL, CCL and SAA signalling pathways are shown. Red fonts highlighted the significant ligand–receptor pairs emanating from DUOX2^+^CEACAM6^+^ enterocytes.

In our clinical trial, UMSC therapy effectively induced clinical remission and colonic mucosal healing in patients. To investigate the mechanism by which UMSC therapy improves the injured mucosal barrier, we analysed the dynamics of the colonic epithelium using scRNA‐seq. We identified 12 subtypes (Figure S4A). Among them, DUOX2^+^CEACAM6^+^ enterocytes and FCGBP^+^TFF3^+^ goblet cells showed the most significant proportion changes throughout therapy (Figure S4B). Dual oxidase 2 (DUOX2) is a hydrogen‐peroxide generator located on the apical membrane of the gastrointestinal epithelia.^35^ DUOX2 was highly expressed in the epithelial cells of the 0‐Mth group, suggesting the potential dysregulation of epithelial cell homeostasis (Figures 5A and S4C). We observed abundant DUOX2 protein in the lateral and perinuclear regions of colonic biopsies from patients with UC. In contrast, DUOX2 was absent in the epithelia of healthy individuals and in the control, 2‐Mth and 6‐Mth groups, indicating that UMSC therapy impeded oxidative damage in enterocytes by inhibiting DUOX2 (Figures 5B and S4D). Additionally, high CEACAM6 expression was notable (Figures 5A and S4C) because it serves as an adhesion molecule that mediates adhesion between bacteria and the host and is highly correlated with IBD.^36^

As enterocytes differentiate from TA cells,^37^ we investigated whether similar functional alterations were present in both TA cells and enterocytes. Following therapeutic intervention, the expression of genes in TA cells decreased to the levels noted in the control group, and enterocytes were predominantly enriched in pathways associated with antigen processing and presentation, cytokine production, oxidative stress and bacterial infection (Figures 5C and S4E). The antigen presentation process in DUOX2^+^CEACAM6^+^ enterocytes, OLFM4^+^ TA cells, and proliferating TA cells was primarily mediated by MHC II genes (e.g., HLA‐DPA1, HLA‐DPB1 and HLA‐DRB5) (Figures 5D and S4F), which are recognised by CD4^+^ T cells to orchestrate immune response against pathogens.^38^ These genes were down‐regulated by UMSC therapy, suggesting that UMSC therapy inhibited excessive immune response by modulating MHC II‐mediated antigen presentation. Additionally, the expression of the pro‐inflammatory genes and chemokines (e.g., CXCL1, CXCL3 and CXCL8) in DUOX2^+^CEACAM6^+^ enterocytes, which are related to the NOD‐like receptor signalling pathway, was reduced after UMSC therapy (Figure 5E).

TA cells differentiate into mature cells, such as goblet cells and enterocytes.^37^ We determined the differentiation patterns of TA cells following UMSC therapy using cell trajectory analysis. As shown in Figures 5F,G and S4G,H, TA cells were predominantly concentrated towards the early pseudotime in the control group, whereas FCGBP^+^TFF3^+^ goblet cells and DUOX2^+^CEACAM6^+^ enterocytes segregated into two distinct branches. Along this trajectory, TA cells were primarily distributed in the early pseudotime, whereas proliferating TA cells and FCGBP^+^TFF3^+^ goblet cells were mainly distributed in the middle to late pseudotime. However, the proportion of TA cells mapped to the early pseudotime decreased in the 0‐Mth group, whereas the proportion of TA cells in branch 3 significantly increased. TA cells exhibited a greater tendency to differentiate towards DUOX2^+^CEACAM6^+^ enterocytes, which were distributed in the middle to late pseudotime period. DUOX2 and CEACAM6 were primarily expressed in cells from branch 3 of the 0‐Mth group. Notably, the proportion of TA cells in the early pseudotime period decreased after treatment. However, their proportion increased in branch 2, but not in branch 3, indicating a tendency to differentiate towards FCGBP^+^TFF3^+^ goblet cells, similar to the control group. Along the trajectory, FCGBP^+^TFF3^+^ goblet cells were primarily distributed from the middle to the right end. To identify changes in the number of goblet cells at the tissue level, we performed alcian blue and periodic acid‐schiff (AB‐PAS) staining and found significant restoration of goblet cells in 2‐Mth and 6‐Mth groups compared with the 0‐Mth group (Figures 5H and S4D). Thus, the epithelial cell dynamics suggest that the inflammatory microenvironment favours the differentiation of TA cells towards DUOX2^+^CEACAM6^+^ enterocytes, and that UMSC therapy induces the differentiation of TA cells towards FCGBP^+^TFF3^+^ goblet cells to enhance immune defence.

### 3.7 UMSC therapy reconstitutes the immune system to protect the mucosal barrier

To elucidate the mechanisms by which UMSC therapy improves the mucosal barrier by modulating immune cells, we investigated the interactions between colonic epithelial cells and immune cells, especially T lymphocytes. The abundance of some cell subtypes was correlated with samples from specific clinical conditions. T cell subsets had a strong positive correlation with DUOX2^+^CEACAM6^+^ enterocytes in the 0‐Mth group, whereas these correlations were markedly diminished in both the 2‐Mth and 6‐Mth groups (Figure S5A). Additionally, we observed a notable decrease in the overall number and strength of interactions between cells in both 2‐Mth and 6‐Mth groups (Figure S5B,C). The most significant decrease in total interaction strength was observed in DUOX2^+^CEACAM6^+^ enterocytes, CD8^+^ TRM, monocytes, macrophages, S100A8^+^ neutrophils and IFIT3^+^ neutrophils (Figure 5I), which serve as the main senders and receivers of all interaction signals (Figure S5D).

We further investigated the interactions between colonic epithelial and immune cells. The CCL, CXCL and SAA signalling pathways were significantly weaker in both 2‐Mth and 6‐Mth groups than in the 0‐Mth group (Figure S5E). Further exploration of the inhibited receptor–ligand pairs identified the interacting molecules as CCL15–CCR1, CCL20–CCR6, CXCL8–CXCR1, CXCL8–CXCR2 and SAA1–FPR2 (Figure 5J). These receptor–ligand pairs were exclusive to the 0‐Mth group (not present in 2‐Mth and 6‐Mth groups), where they primarily mediated the interactions between DUOX2^+^CEACAM6^+^ enterocytes and immune cells (e.g., S100A8^+^ neutrophils, IFIT3^+^ neutrophils, Th17) (Figures 5J and S5F). These findings suggest that UMSC therapy may achieve its regulatory effects on immune cells by inhibiting the CCL, CXCL and SAA signalling pathways in DUOX2^+^CEACAM6^+^ Enterocytes.

### 3.8 UMSCs improve DSS‐induced colitis by regulating T cell‐mediated immunity and inhibiting DUOX2‐mediated oxidative damage in the colonic epithelium

**FIGURE 6:**
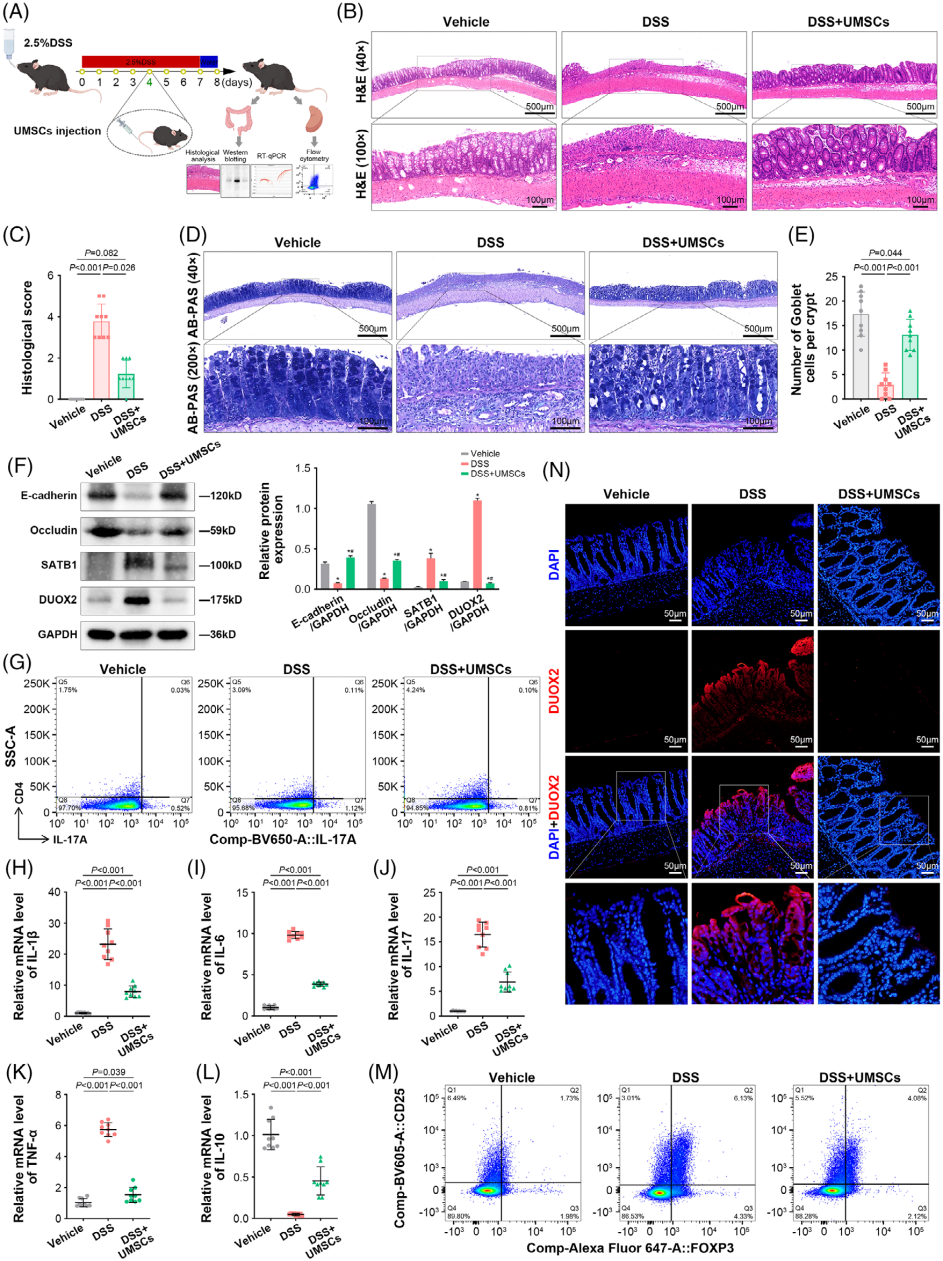
UMSCs improve UC in a DSS‐induced colitis mouse model. (A) Schematic representation of DSS‐induced colitis mouse model experiments with UMSC treatment (male mice, *n* = 9 per group). (B and C) Representative images of H&E staining in colon tissue of the vehicle, DSS and DSS+UMSCs groups (40×, scale bar: 500 µm; 100×, scale bar: 100 µm). Histological scores were used to quantify colon inflammation in the graph. (D and E) Representative images of AB‐PAS staining for goblet cells in the three groups (40×, scale bar: 500 µm; 200×, scale bar: 100 µm). The number of goblet cells is quantified in the graph (right). (F) Representative immunoblots of E‐cadherin, Occludin, SATB1 and DUOX2 of colon tissue in the three groups. Relative protein expression is quantified in the graph (right). ^*^ *p *< 0.05 versus the vehicle group; ^#^ *p *< 0.05 versus the DSS group. (G and M) Representative images of flow cytometry for Th17 and Tregs in the spleen of the three groups. The percentage of Th17 and Tregs is shown in Figure S6F,G. (H–L) Statistical analysis of mRNA levels of IL‐1β, IL‐6, IL‐17, TNF‐α and IL‐10 determined by real‐time PCR analysis in colon tissue of the three groups. (N) Representative images of immunofluorescence (IF) for DUOX2 in colon tissue of the three groups. One‐way ANOVA was used to compare the differences between the three groups.

To validate the effects of UMSC therapy on T cell‐mediated immune and colonic mucosal barrier, we employed a 2.5% DSS‐induced colitis mouse model, then administered UMSCs (10^6^ cells per mouse, *n* = 9) in the tail via intravenous injection (Figure 6A). Compared with the vehicle group, the weight, disease activity index scores and colon length increased in the DSS group but were then reduced by UMSC administration (Figure S6A–C). Haematoxylin and eosin (H&E) and AB‐PAS staining displayed that UMSCs significantly alleviated colonic mucosal injury, reduced histological scores and recovered the number of goblet cells in mice with DSS‐induced colitis (Figure 6B–E). Furthermore, the protein expression of E‐cadherin and Occludin was down‐regulated in DSS‐induced colitis mice; whereas these effects were reversed by UMSCs, suggesting that UMSC therapy protects the mucosal barrier (Figure 6F).

Additionally, flow cytometry and real‐time PCR results showed that the percentage of Th1 and Th17 cells increased in the DSS group, as did mRNA levels of IFN‐γ, IL‐1β, IL‐6, IL‐17 and TNF‐α. In contrast, these effects declined in the DSS+UMSCs group, indicating that UMSC therapy inhibited pro‐inflammatory features in T cells (Figures 6G–K and S6D–F). Notably, despite the high proportion of Tregs, the mean fluorescence intensity of CD39(+) Tregs and IL‐10 mRNA levels were decreased in the DSS group; whereas these effects were reversed by UMSC therapy, suggesting that UMSC therapy restored Treg immunosuppressive function (Figures 6L,M and S6G,H). Furthermore, UMSCs reduced the high SATB1 protein expression induced by DSS (Figure 6F). The results suggest that UMSCs may restore Treg function by inhibiting SATB1 and suppressing the pro‐inflammatory factors secreted from T cells, thereby contributing to immune reconstruction. In addition, western blotting and immunofluorescence showed that elevated DUOX2 expression in the colonic epithelium of DSS‐induced colitis models was down‐regulated by UMSCs, suggesting that UMSC therapy inhibited DUOX2‐mediated oxidative stress (Figure 6F,N).

Hence, UMSC therapy can suppress DUOX2‐mediated oxidative stress to alleviate colonic mucosal injury by reconstituting T cell‐mediated immunity.

## 4 DISCUSSION

To our knowledge, the study elucidates immune reconstitution effects of UMSC therapy against moderate to severe UC via scRNA‐seq. The principal findings were as follows: (1) UMSC infusion effectively induced clinical remission in patients with active left‐sided UC. (2) scRNA‐seq data reveal that UMSC therapy suppressed excessive inflammatory response and restored immune homeostasis by inhibiting the pro‐inflammatory features of T lymphocytes and modulating the interplay between T, B and myeloid cells, contributing to mucosal barrier protection and improvement of enterocyte differentiation. (3) In vivo, UMSCs suppressed DUOX2‐mediated oxidative stress to attenuate DSS‐induced colitis by regulating T cell‐mediated immunity.

The intestinal immune disorder is involved in progression of various gut immunological diseases, such as IBD, intestinal graft‐versus‐host disease (GvHD) and enteropathy in primary immunodeficiency.^39^ Accumulating evidence shows that MSC therapy is beneficial for the maintenance of intestinal immune balance, which is a potential treatment against immunological diseases. In a previous clinical study, MSCs have secured conditional approval for treatment for GvHD in Canada and New Zealand.^40^ In recent years, stem cell therapy, including haematopoietic stem cell and MSC transplantation, has been examined in multiple IBD‐related clinical studies, in which the intestinal immunity and mucosal barrier of patients have been improved effectively.^41^, ^42^ For example, BM‐ and AD‐MSCs promote healing of perianal fistulas in CD.^43^, ^44^, ^45^ Furthermore, UMSC therapy is also a potential treatment for IBD and represents a new area of investigation, owing to its low immunogenicity, more accessibility and immunomodulatory activity.^46^ The infusion or local submucosal injection of UMSCs can safely and effectively improve intestinal ulceration in refractory CD.^15^ Although some case reports or small sample clinical studies have previously reported MSC‐based therapeutic advances for UC, few clinical trials systematically explore the therapeutic efficacy and safety of UMSCs for UC and its underlying mechanisms. Our previous research has confirmed that UMSC infusion (10^6^ cells per kg each month, 2 consecutive months) induces clinical remission of active UC, with no serious adverse events during UMSC therapy or the subsequent 6‐month follow‐up, indicating a safe and effective dosage for active UC (including left‐sided and extensive UC).^21^ In this study, UMSC therapy also effectively induced clinical remission in refractory patients with moderate to severe left‐sided UC, showing significant reductions in erosion, ulceration and inflammatory cell infiltration, as well as mucosal healing in responders from both clinical trials and DSS‐colitis mouse models. Furthermore, the results of plasma inflammatory factors and colonic mucosa scRNA‐seq analysis showed that UMSC therapy may effectively modulate intestinal immunity, thereby attenuating intestinal inflammation in patients with UC. Thus, UMSC therapy shows promise for the clinical application to active UC.

It is crucial to select the optimal dose of UMSCs for treating refractory active UC, which can influence UMSC efficacy directly. However, few clinical studies have reported the influence of UMSC therapeutic efficacy with dose escalation. In recent case reports and clinical trials, the safe dose of UMSC intravenous infusion was reported as approximately 0.5 × 10^6^ to 10^8^ cells for different diseases, such as CD,^15^ liver cirrhosis,^16^ aging frailty,^17^ heart failure,^18^ osteoarthritis^19^ and endometriosis.^20^ The most common dosage of UMSC is 1 × 10^6^ cells/kg administered once a month.^15^, ^17^, ^18^, ^20^ In this study, the dosage (1 × 10^6^ cells/kg, once a month, 2 consecutive months) of UMSCs with intravenous infusion was used for patients with left‐sided UC, which has been reported to be safe and effective for active UC.^21^ Furthermore, we constructed a DSS‐induced acute colitis mouse model and administered the same dosage (1 × 10^6^ cells per mouse) of UMSCs to explore the mechanisms associated with UMSC therapy for UC, and we found that this dosage of UMSCs could improve intestinal inflammation and maintain mucosal barrier integrity. Recent animal experimental evidence reported that UMSCs remarkably attenuated the severity of the disease dose‐dependently in the 2,4,6‐trinitrobenzene sulphonic acid (TNBS)‐induced colitis mouse model.^47^ However, in the clinical trial examining knee osteoarthritis, Jose Matas et al. revealed that the dose escalation (2 × 10^6^ to 80 × 10^6^ cells) of umbilical cord‐derived mesenchymal stromal cell local injection significantly improved clinical features and safety. Moreover, the efficacy of the low (2 × 10^6^ cells) and medium dosage (20 × 10^6^ cells) was better than that of the high dosage (80 × 10^6^ cells).^48^ The therapeutic efficacy of UMSCs seems to differ between animals and patients. Therefore, there is an urgent need to investigate the optimal dose of UMSCs for treating patients with active UC, which will be an important component of our future studies.

However, the mechanisms underlying the efficacy of UMSC treatment in UC remain unclear. In the development of UC, T cell subsets, including Th1, Th2, Th17, Tfh and Treg cells, play pivotal roles in mediating intestinal immunity. Moreover, the activation of T cells and their interaction with other intestinal immune cells, such as B cells and myeloid cells, trigger dysregulated intestinal immunity, thereby driving mucosal inflammation and intestinal impairment.^49^, ^50^ Therefore, targeting T cell‐mediated immunity may be a promising and effective therapy for UC. Given that MSCs primarily act on immune cells to maintain homeostasis,^51^ understanding the role of UMSCs in immune cell modulation, particularly T cell modulation, is essential. Several lines of evidence have revealed the ability of MSCs to suppress T cell proliferation and inflammatory responses in T cells.^52^, ^53^ T cells play a key role in the production and secretion of inflammatory cytokines. Our previous and current research has shown that elevated plasma inflammatory cytokine levels in patients with active UC drastically decreased after UMSC therapy. The current study implied that T cells may be a pivotal target cells for UMSC therapy against UC. Specifically, UMSCs suppressed Th17 cell function by inhibiting activation of the JAK–STAT3 pathway and cytokine production, including IL‐17 production. Furthermore, in a DSS‐induced colitis mouse model, UMSC treatment decreased the proportion of Th1 and Th17 cells and down‐regulated the expression of inflammatory cytokines, including IFN‐γ, IL‐1β, IL‐6, IL‐17, as well as TNF‐α at the mRNA levels. Thus, UMSC therapy may mitigate colonic mucosal injury by suppressing the production of inflammatory cytokines by T cells.

Notably, UMSCs restore the immunosuppressive functions of Tregs. Recent findings demonstrate that a disruption in the Treg/Th17 balance can be attributed to intestinal inflammation^54^, ^55^; however, an increased number of Tregs is noted in the mesenteric lymph nodes and colon lamina propria.^56^, ^57^ SATB1 may be involved in modulating Treg development and maturation.^29^, ^56^ Our scRNA‐seq data and animal experiments showed a high percentage of Tregs with impaired functions in colitis. Nevertheless, UMSCs elevated the levels of CD39 and IL‐10 but reduced SATB1 expression, suggesting that UMSC therapy restored Treg immunosuppressive function by inhibiting SATB1. Additionally, we observed activation of the differentiation pathways of Tfh cells and high expression of IL‐21, which are crucial for the activation of B lymphocytes in GC reactions.^33^, ^58^ Moreover, an increase in Tfh cell numbers and IL‐21 levels in patients with UC is linked to elevate new memory B cells and plasmablasts levels.^59^, ^60^, ^61^ Our scRNA‐seq data showed that UMSC therapy can suppress the activation and migration of Tfh cells by down‐regulating genes (e.g., BCL6, MAF and IL‐21) and inhibiting the CXCR5–CXCL13 axis, contributing to suppression of the excessive humoral immune response. Furthermore, naïve CD4^+^ T cells, which are essential for deriving Tfh and Treg cells, are regulated during gut inflammation. Recent experimental evidence suggests that the differentiation of naïve CD4^+^ T cells towards Tregs is inhibited in DSS‐induced colitis.^56^ Sphingosine 1‐phosphate receptor 1 antagonists promote apoptosis of naïve CD4^+^ T cells and subsequently reduce the number of Tfh and B cells, contributing to suppressing excessive humoral immunity in UC.^62^ However, our scRNA‐seq data showed that UMSC therapy restored the population of naïve CD4^+^ T cells and modulated the differentiation of naïve CD4^+^ T cells into Tregs and Tfh cells. Therefore, we demonstrated that UMSCs suppressed the expression of Th17‐derived IL‐17, inhibited the activation and migration of Tfh and restored the immunosuppressive function of Tregs, aiding in maintaining intestinal immunity homeostasis and suppressing colon inflammation.

Interestingly, CD8^+^ TRM exhaustion was reversed, and the increased expression of IL‐26 was reduced after UMSC therapy. However, the role of IL‐26 in UC remains poorly understood. IL‐26, derived from T cells, plays an important role in mucosal immunity.^32^, ^63^ Our findings suggest that UMSC therapy inhibits CD8^+^ TRM‐derived IL‐26, potentially alleviating epithelial inflammation by suppressing the IL10RB/IL20RA–CXCL8 axis in epithelial cells.

Besides, reliable biomarkers used to evaluate the prognosis of UC and the efficacy of drugs have garnered significant interest. The number of Th17 cells, serum IL‐17 levels and the increased IL‐10/IL‐17 ratio is associated with increased the severity of UC, which may serve as prognostic markers for patients with UC.^64^ The ratios of Treg/Th1, Treg/Th17 and CD8^+^CD28^+^/CD8^+^CD28^−^ T cells have also been used as immunological markers to predict the prognosis of CD and UC.^65^, ^66^ A recent study also identified CD4^+^ T cell‐derived microRNAs as potential biomarkers for evaluating the prognosis and treatment escalation of IBD.^67^ These findings suggest that T cell‐derived products, such as inflammatory factors and microRNAs, contribute to predicting the prognosis of UC and drug efficacy. In our data, UMSC therapy significantly reconstituted T cell‐mediated immunity to improve clinical symptoms and the intestinal mucosal barrier among patients with active UC, suggesting that alterations in T cell subsets may predict and evaluate the therapeutic efficacy of UMSCs in UC. Our previous and current studies showed that plasma levels of IL‐1β, IL‐6, IL‐12 and IL‐17A—cytokines secreted by T cells—were reduced after UMSC therapy, particularly 2‐month post‐therapy in responders,^21^ suggesting that patients showing a reduction in plasma T cell‐derived inflammatory factors may benefit from UMSC therapy. In T cell subsets, we observed an increased number of CD8⁺GZMK^−^ T cells and naïve CD4⁺ T cells, as well as a decreased number of Tfh cells and Tregs in the intestinal mucosa of patients with left‐sided UC following UMSC therapy. More importantly, the differentiation of naïve CD4^+^ T cells into Tregs and Tfh cells was inhibited by UMSCs; moreover, the expression of IL‐21 and SATB1 in Tfh and Treg cells was suppressed at 2 and 6 months post‐therapy. These results suggest that detecting IL‐21 and SATB1 expression, along with the differentiation status of naïve CD4⁺ T cells in the intestinal mucosa, may serve as biomarkers for assessing Tfh cell activation and Treg function, contributing to the evaluation of UC prognosis and UMSC therapeutic efficacy. The interaction between T lymphocytes and other immune cells (e.g., B lymphocytes and myeloid cells) is crucial for modulating immune responses in UC. UMSCs not only suppress pro‐inflammatory features of T cells but also regulate the interaction of T cells with B and myeloid cells. Our data show that UMSC therapy suppresses the JAK–STAT3 pathway and IL‐17 production in Th17 cells and reverses the excessive activation of B cells by inhibiting the Tfh cell‐mediated CXCR5–CXCL13 axis, thereby improving immune dysregulation in UC. This effect is similar to the immune improvements observed following Ustekinumab and Tofacitinib treatments.^23^, ^68^ Myeloid cell function is regulated by T cells, especially Tregs. A growing literature shows that Tregs modulate the differentiation and functions of neutrophils and macrophages, as well as the apoptosis of monocytes, to improve the inflammatory response.^69^, ^70^, ^71^ In this research, UMSCs reduced the infiltration of neutrophils and monocytes by suppressing signalling pathways and the CXC6–CXCL6 axis played an important role in Tregs and myeloid cells (e.g., neutrophils and monocytes), thereby inhibiting the spread of inflammation.

Finally, we focus on colonic epithelial cells that interact with immune cells to protect mucosal barrier function. DUOX2 overexpression leads to the excessive generation of reactive oxygen species and reduces mucin 2 in IBD.^72^ Our scRNA‐seq data showed that UMSC therapy significantly reduced DUOX2 expression and recovered the number of goblet cells by inhibiting the differentiation of TA cells into DUOX2^+^CEACAM6^+^ enterocytes. These findings in patients were confirmed by the animal model of DSS‐induced colitis treated with UMSCs. However, the molecular mechanisms underlying the attenuation of mucosal inflammation by UMSCs remain unclear. Recent evidence suggests that MSC infusion maintains mucosal barrier integrity by enhancing the circulating IGF‐1 level instead of directly affecting the impaired mucosa.^9^ Our findings showed that UMSC infusion effectively inhibited the recruitment of immune cells by suppressing signalling pathways such as CCL20–CCR6, CXCL8–CXCR1/CXCR2 and SAA–FPR2 in DUOX2^+^CECAM6^+^ enterocytes, thereby rebuilding the immune system to protect the mucosal barrier.

This study still has some limitations. In this small‐sample, single‐arm clinical trial, we enrolled 26 patients with moderate to severe left‐sided UC who received UMSC therapy and conducted a 6‐month follow‐up to explore the therapeutic efficacy of UMSCs and the potential underlying mechanisms of UMSCs. Furthermore, the larger sample size of multicentre, randomised controlled trials are urgently needed to confirm the efficacy and safety of UMSCs for active UC. Moreover, combining UMSC therapy with other treatments (such as biological agents, small molecule therapies and faecal microbiota transplantation) may represent a promising approach for treatment of UC, which warrants further investigation. Additionally, UMSCs may simultaneously modulate the functions of various cells (including T cells, B cells, myeloid cells and epithelial cells). To explore the roles of UMSC‐regulated T cells in relation to other immune and epithelial cells, future studies should further validate the specific key pathways governing the interactions of T cells with other immune cells and epithelial cells in the context of UMSC therapy against gut inflammation.

In summary, the results of our clinical trials and animal experiments elucidate the mechanisms of UMSC therapy in UC. Principally, UMSC therapy modulates T cell‐mediated immunity to reconstitute the immune system, thereby maintaining mucosal barrier integrity. The therapeutic effects of UMSC suggest potential against a range of immune‐related disorders, including UC, and provide valuable insights for further research and clinical applications.

## AUTHOR CONTRIBUTIONS

X. L., X. J., Y. B., H. Z., H. D., Z. Y., Y. W., Z. L., L. L. and L. Z. were responsible for patient enrolment and biopsy sample acquisition. Y. L. conducted UMSCs preparation for the clinical trial and animal experiments. J. D. and J. M. conducted the data analysis and interpretation, with support from B. Z., O. J. L., H. Y. and G. C. X. L., M. L., C. C., P. L., H. H., Y. X. and Y. Q. executed functional validation experiments. Y. M. and L. W. processed samples for scRNA‐seq. X. L., J. D. and X. J. drafted the manuscript. All co‐authors reviewed and approved the final version of the manuscript.

## CONFLICT OF INTEREST STATEMENT

The authors declare that they have no conflicts of interest.

## ETHICS STATEMENT

The study was approved by the Committee on the Ethics of Henan Provincial People's Hospital (approval number: (2018) NO. 03‐01). The mouse experiments were approved by the Committee on the Ethics of Animal Experiments of Zhengzhou University (Zhengzhou, China) (ethical approval code ZZU‐LAC20220422[16])

## CODE AVAILABILITY

All code written and utilised in this study is available from the corresponding author upon reasonable request

## Supporting information



Supporting Information

Supporting Information

Supporting Information

Supporting Information

Supporting Information

## Data Availability

All data needed to evaluate the conclusions in the paper are present in the paper and/or the Supporting Information. The raw sequence data reported in this paper have been deposited in the Genome Sequence Archive (https://ngdc.cncb.ac.cn/gsa/; GSA‐Human: HRA006099).
