# Supplementary material for: Reconstitution of T cell‐mediated immunity by umbilical cord‐derived mesenchymal stem cells in ulcerative colitis
Source: Clin Transl Med. 2025 Aug 21;15(8):e70452. doi: 10.1002/ctm2.70452 (PMC12371211; doi:10.1002/ctm2.70452)
Supplement: Supplementary file 1 — Supporting Information [file CTM2-15-e70452-s005.docx]

**Supplement**

**1 Authors information**

**Xiaoying Luo^#^**, Department of Gastroenterology, Microbiome Laboratory, Henan Provincial People's Hospital, People's Hospital of Zhengzhou University, Zhengzhou University, Zhengzhou, 450000, China. E-mail: [lxycs2015@zzu.edu.cn](mailto:lxycs2015@zzu.edu.cn)

**Jieping Deng^#^**, Department of Systems Biomedical Sciences, School of Medicine, Jinan University, Guangzhou, 510000, China. E-mail: [djieping@163.com](mailto:djieping@163.com)

**Xiaoke Jiang^#^**, Department of Gastroenterology, Henan Provincial People's Hospital, People's Hospital of Zhengzhou University, Zhengzhou University, Zhengzhou, 450000, China. E-mail: [xiaoke02156@163.com](mailto:xiaoke02156@163.com)

**Jun Mi**, Department of Gastroenterology, Henan Provincial People's Hospital, People's Hospital of Zhengzhou University, Zhengzhou University, Zhengzhou, 450000, China. E-mail: [mijun0720@126.com](mailto:mijun0720@126.com)

**Yangqiu Bai**, Department of Gastroenterology, Henan Provincial People's Hospital, People's Hospital of Zhengzhou University, Zhengzhou University, Zhengzhou, 450000, China. E-mail: [doctbai@126.com](mailto:doctbai@126.com)

**Huimin Zhang**, Department of Gastroenterology, Henan Provincial People's Hospital, People's Hospital of Zhengzhou University, Zhengzhou University, Zhengzhou, 450000, China. E-mail: [huiminzhang91@163.com](mailto:huiminzhang91@163.com)

**Yalong Li**, Stem Cell Research Center, Henan Key Laboratory of Stem Cell Differentiation and Modification, Henan Provincial People's Hospital, People's Hospital of Zhengzhou University, Zhengzhou University, Zhengzhou, 450000, China. E-mail: [liyalong0718@126.com](mailto:liyalong0718@126.com)

**Min Liu**, Department of Gastroenterology, Microbiome Laboratory, Henan Provincial People's Hospital, People's Hospital of Zhengzhou University, Zhengzhou University, Zhengzhou, 450000, China. E-mail: [liumin1120@gs.zzu.edu.cn](mailto:liumin1120@gs.zzu.edu.cn)

**Conghui Cai**, Department of Gastroenterology, Microbiome Laboratory, Henan Provincial People's Hospital, People's Hospital of Zhengzhou University, Zhengzhou University, Zhengzhou, 450000, China. E-mail: [cch184379078711103@163.com](mailto:cch184379078711103@163.com)

**Pengju Li**, Department of Gastroenterology, Luoyang Central Hospital Affiliated to Zhengzhou University, Luoyang, 471000, China. E-mail: [lipengjuvvvip@163.com](mailto:lipengjuvvvip@163.com)

**Huanrong Huang**, Department of Gastroenterology, Puyang People’s Hospital, Puyang, 457005, China. E-mail: [hhrong1202@gs.zzu.edu.cn](mailto:hhrong1202@gs.zzu.edu.cn)

**Yueping Xu**, Department of Gastroenterology, Microbiome Laboratory, Henan Provincial People's Hospital, People's Hospital of Zhengzhou University, Zhengzhou University, Zhengzhou, 450000, China. E-mail: [x13663710183@gs.zzu.edu.cn](mailto:x13663710183@gs.zzu.edu.cn)

**Yiwen Qin**, Department of Gastroenterology, Microbiome Laboratory, Henan Provincial People's Hospital, People's Hospital of Zhengzhou University, Zhengzhou University, Zhengzhou, 450000, China. E-mail: [qinyiwen@gs.zzu.edu.cn](mailto:qinyiwen@gs.zzu.edu.cn)

**Yang Mi**, Key Laboratory of Helicobacter pylori & Microbiota and Gastrointestinal Cancer of Henan Province, Marshall Medical Research Center, The Fifth Affiliated Hospital of Zhengzhou University, Zhengzhou, 450000, China. E-mail: [yangmi198@zzu.edu.cn](mailto:yangmi198@zzu.edu.cn)

**Hui Ding**, Department of Gastroenterology, Henan Provincial People's Hospital, People's Hospital of Zhengzhou University, Zhengzhou University, Zhengzhou, 450000, China. E-mail: [dinghui76sy@163.com](mailto:dinghui76sy@163.com)

**Zhiyu Yang**, Department of Gastroenterology, Henan Provincial People's Hospital, People's Hospital of Zhengzhou University, Zhengzhou University, Zhengzhou, 450000, China. E-mail: [back99999@126.com](mailto:back99999@126.com)

**Yue Wu**, Department of Gastroenterology, Henan Provincial People's Hospital, People's Hospital of Zhengzhou University, Zhengzhou University, Zhengzhou, 450000, China. E-mail: [c_zzmay@qq.com](mailto:c_zzmay@qq.com)

**Zhenjuan Li**, Department of Gastroenterology, Henan Provincial People's Hospital, People's Hospital of Zhengzhou University, Zhengzhou University, Zhengzhou, 450000, China. E-mail: [sunnygirl_1205@163.com](mailto:sunnygirl_1205@163.com)

**Ling Lan**, Department of Gastroenterology, Henan Provincial People's Hospital, People's Hospital of Zhengzhou University, Zhengzhou University, Zhengzhou, 450000, China. E-mail: [lanling95@163.com](mailto:lanling95@163.com)

**Lida Zhang**, Department of Gastroenterology, Heart Center of Henan Provincial People's Hospital, Central China Fuwai Hospital of Zhengzhou University, Zhengzhou, 450000, China. E-mail: [zhanglida2002@zzu.edu.cn](mailto:zhanglida2002@zzu.edu.cn)

**Li Wang**, Henan Academy of Innovations in Medical Sciences, Zhengzhou, 450000, China. E-mail: [hnwl65588708@163.com](mailto:hnwl65588708@163.com)

**Guobing Chen**, Department of Microbiology and Immunology, Institute of Geriatric Immunology, School of Medicine, Jinan University, Guangzhou, 510000, China. E-mail: [guobingchen@jnu.edu.cn](mailto:guobingchen@jnu.edu.cn)

**Han Yue**, Stem Cell Research Center, Henan Key Laboratory of Stem Cell Differentiation and Modification, Henan Provincial People's Hospital, People's Hospital of Zhengzhou University, Zhengzhou University, Zhengzhou, 450000, China. E-mail: [Yuehan1000@126.com](mailto:Yuehan1000@126.com)

**Oscar Junhong Luo^*^**, Department of Systems Biomedical Sciences, School of Medicine, Jinan University, Guangzhou, 510000, China. E-mail: [luojh@jnu.edu.cn](mailto:luojh@jnu.edu.cn)

**Bingying Zhang^*^**, Department of Gastroenterology, Henan Provincial People's Hospital, People's Hospital of Zhengzhou University, Zhengzhou University, Zhengzhou, 450000, China. E-mail: [zhbingyong@sina.com](mailto:zhbingyong@sina.com)

^#^These authors contributed equally: Xiaoying Luo, Jieping Deng, Xiaoke Jiang

^*^Correspondence author: Prof. Han Yue, Prof. Oscar Junhong Luo, Prof. Bingyong Zhang

**2 Funding information**

The work is supported by funding from the major program of provincial and ministerial co-construction in medical science and technique foundation of Henan Province (SBGJ202001002); the National Health Commission project (CMR-20180615-1001); the Key R&D and Promotion Project of Henan Province (Technology Research) (232102311040); the Pearl River Talents Scheme of Guangdong Province (2019QN01Y990); Guangdong Basic and Applied Basic Research Foundation (2023B1515040016).

**3 Supplementary methods**

**3.1 Preparation of Human UMSCs**

Umbilical cord samples from three full-term healthy male infants were collected, with informed consent from donor. The study was approved by the Committee on the Ethics of Henan Provincial People's Hospital (approval number: (2018) NO. 03-01). Subsequently, the UMSCs extracted from the umbilical cords were isolated and identified according to our previous methods^1^. UMSCs were cultured in serum-free medium (#NC0106, Yocon, China) in the cell incubator with 5% CO_2_ at 37℃. Similar to previous studies^2^, the third generation of UMSCs were used in the subsequent therapy.

**3.2 Immunohistochemistry (IHC) for DUOX2 in colonic biopsy specimens of UC patients**

Ascending colonic and sigmoid colonic biopsy specimens were obtained from 26 left-sided UC patients, while normal sigmoid colonic biopsy specimens were obtained from 26 healthy individuals. Immunohistochemical detection of DUOX2 (1:100, Santa Cruz, sc-398681, USA) were performed on paraffin sections (4μm) of colonic biopsy specimens, and subsequent sections were exposed to HRP-antibody, colored with DAB. The sections were visualized by microscopy (BX51, Olympus; Japan).

**3.3 Detection of plasma IL-1β, IL-6, IL-8, IL-12, and IL-17A in UC patients**

The plasma IL-1β, IL-6, IL-8, IL-12, and IL-17A of the enrolled individuals (n=26) and the age and gender-matched healthy controls (n=26) were measured by Human IL-1β ELISA Kit (rx106152h, Quanzhou Ruixin Biological Technology Co., LTD., Quanzhou, China), Human IL-6 ELISA Kit (rx106126h, Quanzhou Ruixin Biological Technology Co., LTD., Quanzhou, China), Human IL-8 ELISA Kit (rx106123h, Quanzhou Ruixin Biological Technology Co., LTD., Quanzhou, China), Human IL-12 ELISA Kit (rx106165h, Quanzhou Ruixin Biological Technology Co., LTD., Quanzhou, China), and Human IL-17A ELISA Kit (rx106159h, Quanzhou Ruixin Biological Technology Co., LTD., Quanzhou, China), according to the manufacturer's instructions and subsequently read by ELISA.

**3.4 Colonic mucosal sample processing and library preparation for scRNA-seq**

According to the clinical response rate (75%) at six months post-therapy in this clinical trial, of the 26 patients with moderate to severe left-sided UC, we selected eight patients’ colonic mucosal samples for scRNA-seq. We collected total 22 colonic mucosal samples from the eight patients, six of which were responders and two of which were non-responders. There were 8 samples from clinically non-inflamed sites (ascending colon, namely Ctrl, n=8) and 8 samples from inflamed sites (sigmoid colon, namely 0-Mth) in the eight patients prior to UMSC therapy. After UMSC treatment for comparison, we collected 2 samples from previously inflamed sites (sigmoid colon, namely 2-Mth) at two months post-therapy and 4 samples from previously inflamed sites (sigmoid colon, namely 6-Mth) at six months post-therapy (Supplementary Figure 1A–B).

We transferred human intestinal mucosal tissue to a culture dish with 10 mL of RPMI 1640 medium, removing any blood clots, necrotic tissue, fascia, and other non-target tissues and impurities, and then cut the tissue into small pieces and transferred them to a 15 mL centrifuge tube with 5 mL of digestion solution. The tissue was digested at 37°C for 30-40 minutes with periodic agitation. We added 5 mL of pre-cooled RPMI 1640 medium to stop digestion. The sample was filtered through a 70 µm strainer and centrifuged at 4°C, 300×g for 5 minutes. The cells were resuspended in 1×PBS, and after another centrifugation, and adjusted the volume for further use. Cell viability was confirmed above 85%, and clumping was below 5%. The cell count was at least 0.2 million before proceeding with scRNA-seq library preparation (Doc ID: 210964 Rev. 1.0). On average, 30,000 pooled cells were loaded into each cartridge on the BD Rhapsody^TM^ Express Single Cell Analysis System for single cell capture. This was followed by cDNA synthesis in accordance with the manufacturer's guidelines (Doc ID: 210967 Rev. 1.0). Once the cells are captured on the BD Rhapsody^TM^ Express Single Cell Analysis System, generate a single-cell whole transcriptome mRNA library using the mRNA Whole Transcriptome Analysis (WTA) Library Preparation Protocol (Doc ID: 23-21711-00). Finally, the libraries were sequenced on Illumina NovaSeq 6000 platform.

**The inflammation signature score**

To validate our endoscopic assessments of tissue inflammation, we established an inflammation score by evaluating the expression of the following genes: IFNG, IFNGR1, IFNGR2, IL10, IL12A, IL12B, IL12RB1, IL12RB2, IL13, IL17A, IL17F, IL18, IL18R1, IL18RAP, IL1A, IL1B, IL2, IL21, IL21R, IL22, IL23A, IL23R, IL2RG, IL4, IL4R, IL5, IL6, JUN, NFKB1, RELA, RORA, RORC, S100A8, S100A9, STAT1, STAT3, STAT4, STAT6, TGFB1, TGFB2, TGFB3, TNF, and FOXP3. The P-value for the comparison between the inflammatory group and the other three groups were calculated using the Wilcoxon test (^*^*P*<0.05; ^**^*P*<0.01; ^***^*P*< 0.001).

**GO and KEGG signaling pathway enrichment analysis**

Gene Ontology (GO) and Kyoto Encyclopedia of Genes and Genomes (KEGG) pathway enrichment analysis were performed using the clusterProfiler package ^3^ (v.4.7.1.003) in the R software. Visualization of the analysis results was achieved with the ggplot2 package (v.3.4.2). The key parameter configurations were as follows: enrichment significance threshold (*p*-value)<0.05 and adjusted p-value (q-value)<0.05. All enrichment results are listed in Table S4.

**Cell fate trajectory analysis**

The R package Monocle ^4^ (v.2.22.0) was used for cell trajectory and pseudotime analyses for selected cell clusters. Briefly, we combined DE genes from various cell types and employed DDRTree and pseudotime ordering to further analyze the relationships among these cell types. To identify genes with expression patterns that correlate with pseudotime, we computed Spearman correlations between the predicted pseudotime and the gene expression levels of cells. Genes displaying an absolute Spearman correlation coefficient (p)> 0.5 were extracted. The expression levels of these genes were normalized through z-score transformation and smoothed for heatmap visualization (R package pheatmap, v.1.0. 12).

**Cell communication analysis**

To analyze intercellular communication, we employed the R package CellChat ^5^ (v.1.6. 1). Briefly, CellChat objects were created with the function createCellChat. Communication probabilities between cell subsets were calculated to predict the communication network. The pathway signal strength across different groups were showed via function netVisual_diffInteraction. We identified and visualized conservative and specific signaling pathways using function rankNet. The number of interactions and network centrality were handled with function netVisual_circle and netAnalysis_computeCentrality. Differences in interaction were showcased with function netVisual_heatmap, and significant ligand-receptor interactions were highlighted using function netVisual_bubble.

**Cell cycle scoring**

Utilizing the expression patterns of classical marker genes associated with the G2/M and S phases, we derived scores to determine the likely cell cycle stage of each individual cell. These marker gene sets exhibit an inverse correlation with their expression levels, and cells devoid of expression of these markers are typically presumed to be in the G1 phase. The function CellCycleScoring is employed to compute these cell cycle scores for each cell. Subsequently, the calculated scores for the S and G2/M phases are stored in the metadata, along with the classification indicating whether the cell is likely in the G2/M, S, or G1 phase. By enabling the set.ident parameter to be true, the function CellCycleScoring assigns grouping information to each cell within the Seurat object based on its cell cycle stage.

**Single-cell regulatory network inference**

To deduce the regulatory activity of transcription factors within myeloid-cell subsets, we used the pySCENIC ^6^ package (v.0.12. 1) for gene regulatory network analysis. Briefly, single-cell gene expression count matrix from myeloid cells (neutrophils, monocytes, macrophages, and DCs) within each group and a list of 1,839 known human transcription factors (<https://scenic.aertslab.org/>) were firstly fed into SCENIC. Next, we employed the GRNBoost algorithm to discern co-expression connections between transcription factors and potential target genes, thereby creating co-expression modules. Subsequently, we used RcisTarget for DNA motif analysis, weeding out false positives and indirect targets, and culminating in the identification of direct target genes for each transcription factor, forming a regulon. Finally, the AUCell was used to analyze the activity of each cell's regulon, scoring it based on gene expression values. This process enabled us to pinpoint cells with markedly elevated regulon activity and convert these scores into a binary matrix.

**Histological analysis and AB-PAS staining**

Paraffin sections (4μm) of colonic biopsy specimens from 26 left-sided UC patients and 26 healthy individuals, as well as mouse colon tissues in the three groups were prepared with hematoxylin and eosin (H&E) staining and the AB-PAS staining. The sections were visualized by microscopy (BX51, Olympus, Japan).

**Immunofluorescent staining for DUOX2 in mouse colon tissue**

Paraffin sections (4μm) of mouse colon tissues in the three groups were prepared for immunofluorescent staining, incubated with anti-DUOX2 (1:100, Santa Cruz, sc-398681, USA) overnight, followed by Cy3-labeled goat anti-rabbit IgG (H+L) (1:200, Beyotime, A0516, China). The sections were visualized by luminescence microscope.

**Western blotting**

The protein expression in colon tissue of mouse models was detected by western blot. The primary antibodies included anti-E-cadherin (1:1000, Proteintech, 20874-1-AP, China), anti-Occludin (1:1000, Proteintech, 66378-1-ig, China), anti-SATB1 (1:1000, Proteintech, 15400-1-AP, China), anti-DUOX2 (1:1000, Santa Cruz, sc-398681, USA), and anti-GAPDH (1:1000, Zenbio, 380626, China). The secondary antibodies included Goat anti-mouse IgG H&L (HRP) (1:10000, Zenbio, 511103, China) and Goat anti-rabbit IgG H&L (HRP) (1:10000, Zenbio, 511203, China). The protein bands were detected with Pierce^TM^ ECL Western Blotting Substrate and were quantified by Gel-Pro analyzer 4.0.

**RT-qPCR**

Total RNA of colon tissues in mouse models was extracted with TRIZOL Reagent (Sigma-Aldrich) and reverse transcribed with a NovoScript® Plus All-in –one 1^st^ Strand cDNA Synthesis SuperMix (gDNA Purge) Kit (Novoprotein Scientific Inc., E047, China). The mRNA expression was detected by Real-time PCR. The samples were analyzed using the 2^-ΔΔCT^ method from the Ct values. The primers included the following: IL-1β fwd 5’-TGGACCTTCCAGGATGAGGACA-3’ and rev5’-GTTCATCTCGGAGCCTGTAGTG-3’; IL-6 fwd 5’-TAGTCCTTCCTACCCCAATTTCC-3’ and rev5’-TTGGTCCTTAGCCACTCCTTC-3’; IL-17 fwd 5’-TTTAACTCCCTTGGCGCAAAA-3’ and rev5’-CTTTCCCTCCGCATTGACAC-3’; TNF-α, fwd 5’-TCCCCAAAGGGATGAGAAGTT-3’ and rev5’-GAGGAGGTTGACTTTCTCCTGG-3’; IL-10, fwd 5’-TACAGCCGGGAAGACAATAACT-3’ and rev5’- AGGAGTCGGTTAGCAGTATGTTG-3’; IFN-γ fwd 5’-CAGCAACAGCAAGGCGAAAAAGG-3’ and rev5’- TTTCCGCTTCCTGAGGCTGGAT-3’; GAPDH fwd 5’-AGGTCGGTGTAACGGATTTG-3’ and rev5’- TGTAGACCATGTAGTTGAGTCA-3’.

**
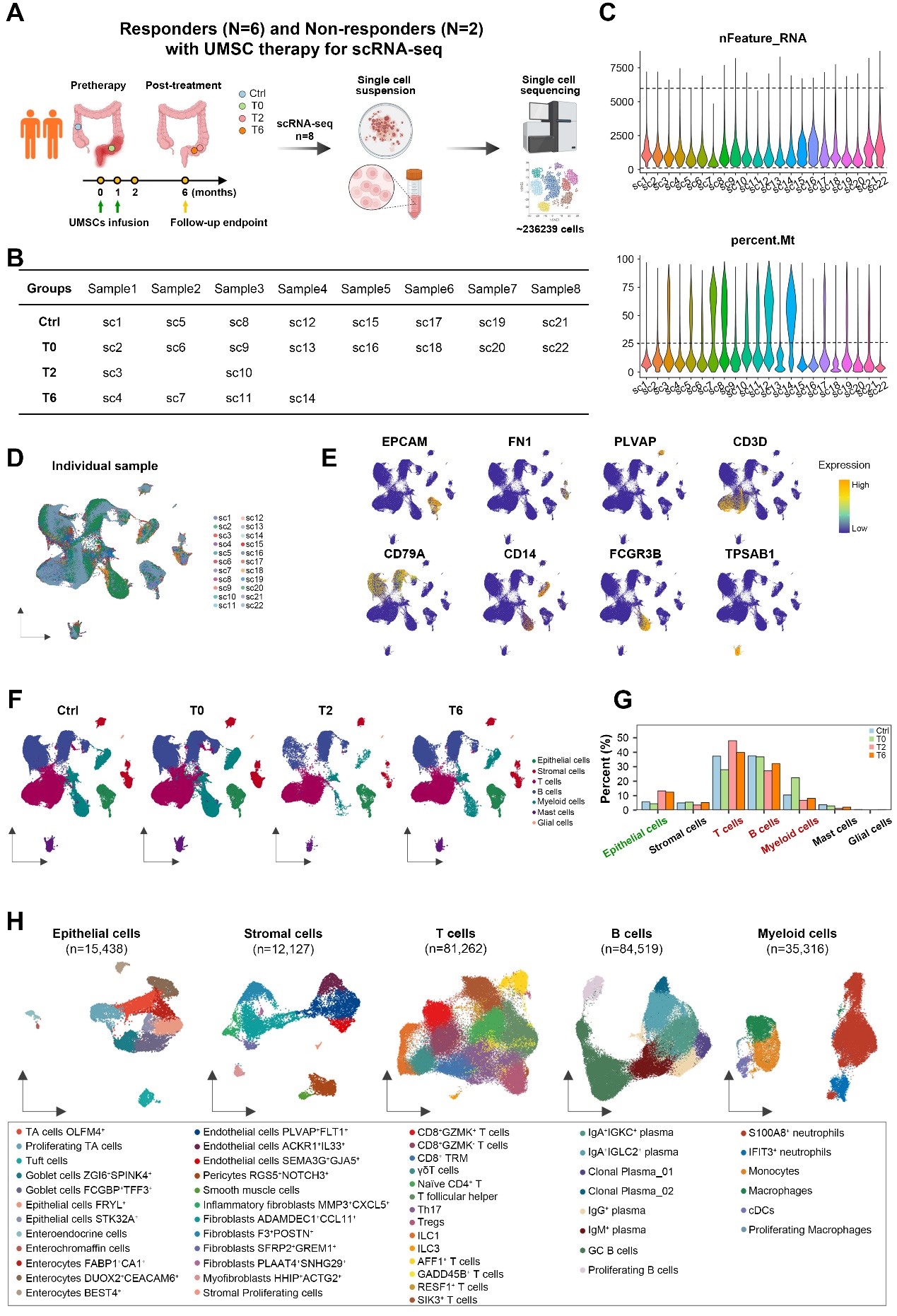
Supplementary Figure legends**

**Figure S1 Single-cell composition on colonic biopsy specimens of active left-sided UC patients at pre-therapy and post-therapy.** Total 22 colonic mucosal samples were collected from 8 patients (including 6 responders and 2 non-responders) for scRNA-seq at pre-therapy, two and six months post-therapy. (**A**) Schematics of colon biopsy specimens and scRNA-seq. (**B**) Information of scRNA-seq samples (Ctrl, n=8; T0, n=8; T2, n=2; T6, n=4). (**C**) Violin plots of unique genes (Up) and percentage of mitochondria genes (Down) detected by scRNA-seq data in each sample. The horizontal dashed lines in the up panel mark 200 and 5000, respectively. The horizontal dashed line in the down panel marks 25%. Mt stands for mitochondria. (**D**) UMAP plot of all single cells colored by samples. The UMAP plot shows no obvious sample bias or batch effect in the scRNA-seq data. (**E**) Projection of expression level of selected marker gene on UMAP in **D**. (**F**) UMAP plots of scRNA-Seq data split by sample groups. (**G**) Bar plot illustrating the proportion of cell subsets. (**H**) UMAP plots of each major cell-type (epithelial, stromal, T, B, and myeloid cells) colored by detailed subtypes.


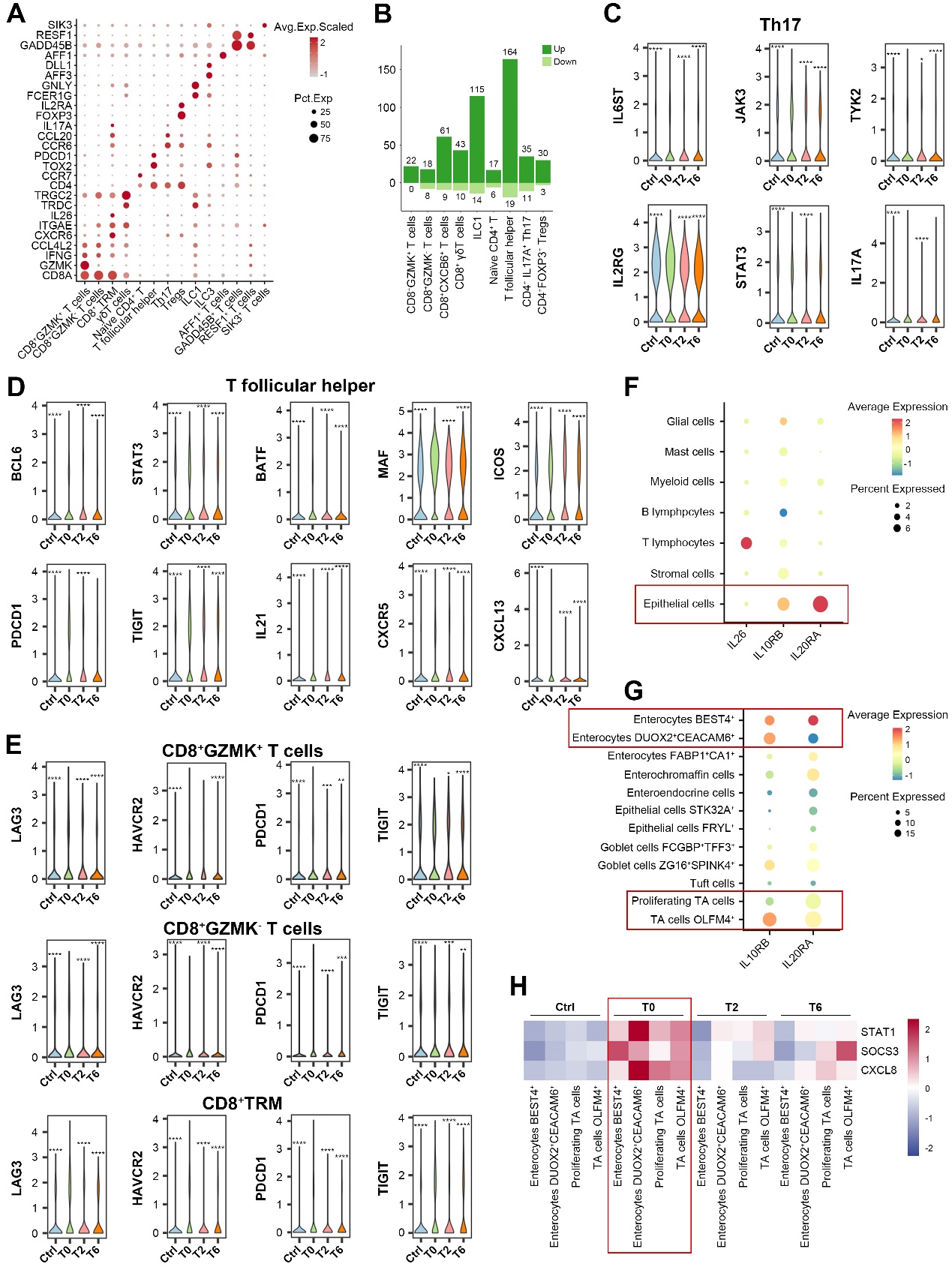


**Figure S2** **Changes in gene expression, signaling pathways, and cell-cell interactions in T lymphocytes before and after UMSC treatment.** (**A**) Dot plot of marker genes for T lymphocyte subtypes. (**B**) Bar plot showing the counts of up-regulated and down-regulated genes within T lymphocytes that were altered after UMSC therapy. (**C to E**) Violin plots illustrating significant differences in T cell-specific genes within the four groups (Ctrl, T0, T2, and T6). Pairwise comparisons were performed between the T0 group and the other three groups (Wilcoxon test, ^*^*P*<0.05, ^**^*P*<0.01, and ^***^*P*<0.001). (**F** and **G**) Dot plots illustrating the normalized expression levels of IL26, along with its receptors IL10RB and IL20RA, across seven cell types (**F**) and within epithelial cell clusters (**G**). (**H**) Heatmap of average scaled expression of STAT1, SOCS3 and CXCL8 within TA cell, proliferating TA cell, DUOX2^+^CEACAM6^+^ and BEST4^+^ enterocyte clusters across the four sample groups. The red rectangle highlights high gene expression in these cell types.


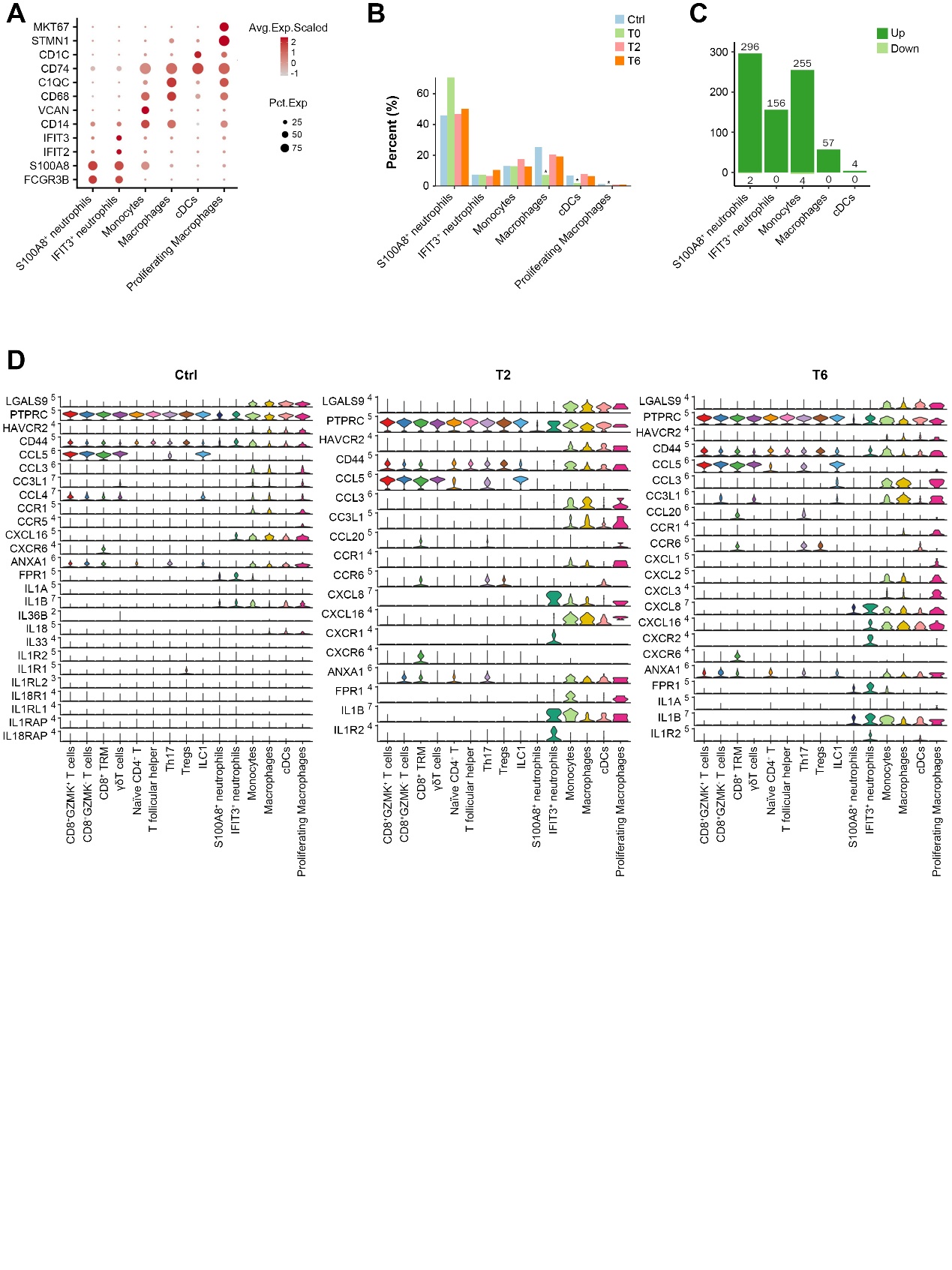


**Figure S3 Modulation of myeloid cells subsets and cell-cell interactions between T lymphocytes and myeloid cells following UMSC treatment.** (**A**) Dot plot of marker genes for cell subtypes within myeloid cells. (**B**) Bar plots of proportions of different myeloid cell subsets among the four groups (Ctrl, T0, T2, and T6). See Table S3. Asterisks indicated significant differences in pairwise comparisons (Wilcoxon test; ^*^*P*<0.05, ^**^*P*<0.01, and ^***^*P*<0.001). (**C**) Bar plot illustrating the counts of up-regulated and down-regulated genes within myeloid cells that were altered after UMSC therapy. (**D**) Violin plots of normalized expression of key ligand-receptor genes between T lymphocytes and myeloid cells in the Ctrl, T2, and T6 groups.


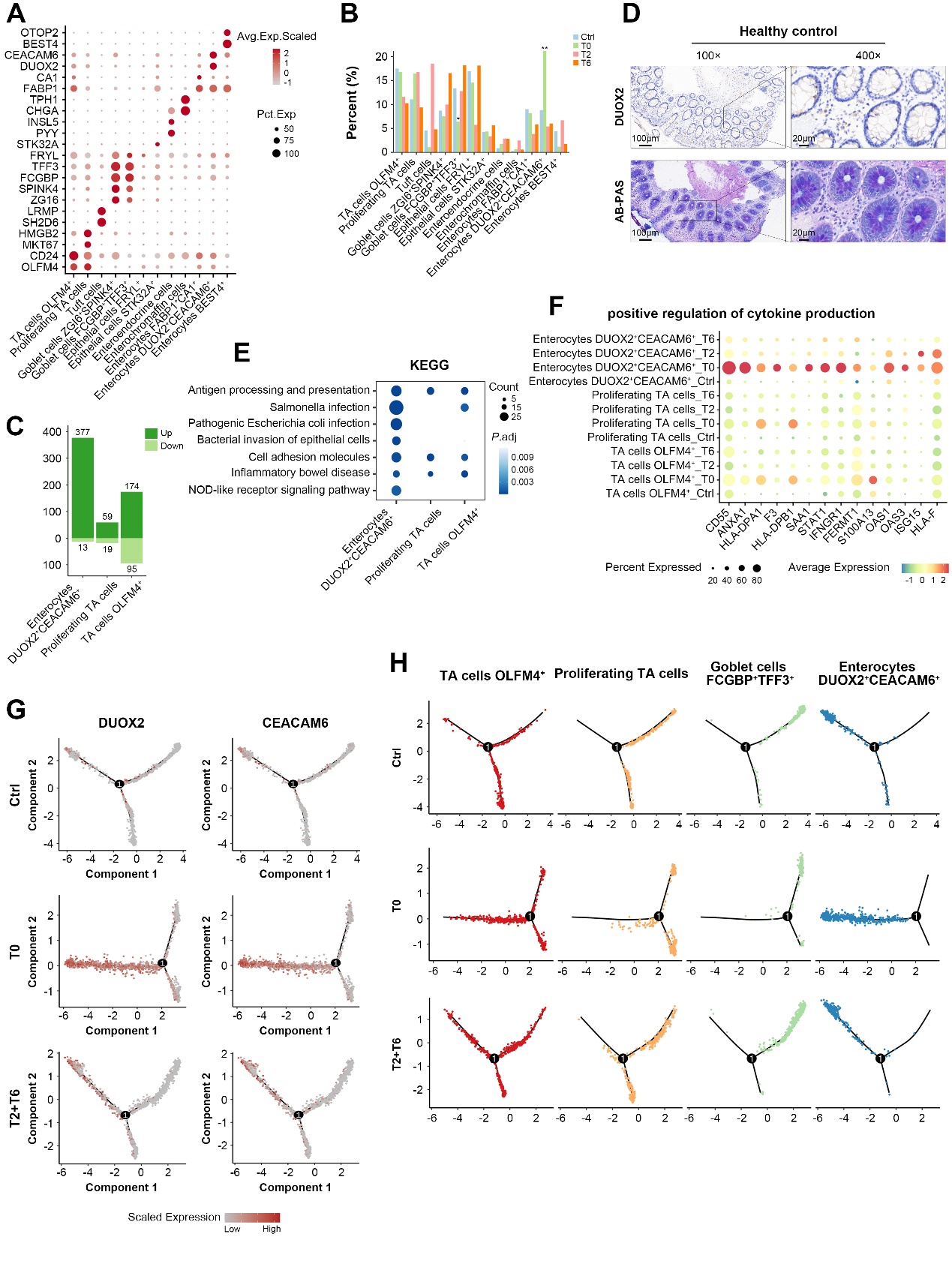


**Figure S4 Alterations in gene expression, signaling pathways, and cell differentiation of epithelial cells at pre-therapy and post-therapy.** (**A**) Dot plot of marker genes for cell subtypes within the epithelial cells. (**B**) Bar plots of proportions of different epithelial cell subsets among the four groups (Ctrl, T0, T2, and T6). See Supplementary Table 3. Asterisks indicated significant differences in pairwise comparisons (Wilcoxon test; ^*^*P*<0.05, ^**^*P*<0.01, and ^***^*P*<0.001). (**C**) Bar plot illustrating the counts of upregulated and downregulated genes within DUOX2^+^CEACAM6^+^ enterocytes, proliferating TA and OLFM4^+^ TA cells that were altered after UMSC therapy. (**D**) Representative images of immunohistochemistry (IHC) for DUOX2 and AB-PAS staining for goblet cells in colonic biopsy specimens of healthy control group (n=26) (100×, scale bar: 100μm; 400×, scale bar: 20μm). (**E**) KEGG pathways that showed significant enrichment for the upregulated genes for DUOX2^+^CEACAM6^+^ enterocytes, proliferating TA, and OLFM4^+^ TA cells in the T0 group. Differential gene expression was compared between T0 and Ctrl groups, as well as between T0 and combined T2+T6 groups. KEGG pathway enrichment analysis focused on the common upregulated genes. (**F**) Dot plot illustrating the normalized expression of genes enriched in the GO terms of positive regulation of cytokine production within DUOX2^+^CEACAM6^+^ enterocytes, proliferating TA, and OLFM4^+^ TA cells across the four groups (Ctrl, T0, T2, and T6). (**G**) Projection of DUOX2 and CECAM6 expression levels on cell trajectories of TA cells, FCGBP^+^TFF3^+^ goblet cells, and DUOX2^+^CEACAM6^+^ enterocytes across the Ctrl, T0, and combined T2+T6 groups. (**H**) Distribution of TA cells, FCGBP^+^TFF3^+^ goblet cells and DUOX2^+^CEACAM6^+^ enterocytes in each branch of the differentiation trajectories.


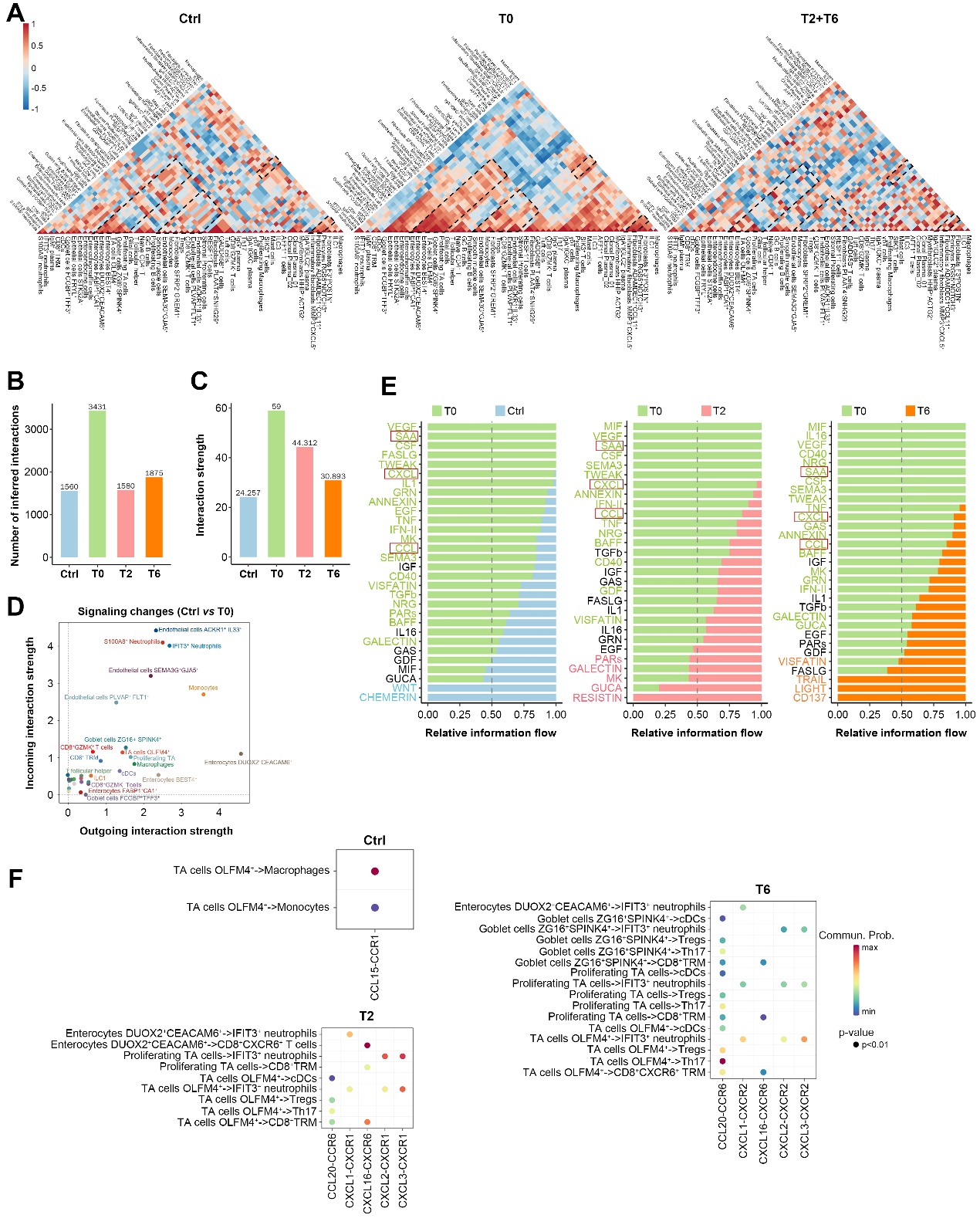


**Figure S5 Changes in cell abundance correlation and alterations in cell-cell interactions before and after UMSC treatment.** (**A**) Heatmaps depicting the correlation of cell type proportions within the Ctrl, T0, and combined T2+T6 groups. (**B** and **C**) Bar plots showing the number (**B**) and strength (**C**) of interaction between epithelial, endothelial, T, and myeloid cells across the four groups (Ctrl, T0, T2, and T6). (**D**) Scatter plot depicting differential signaling roles of each cell cluster in signaling pathways when comparing the Ctrl group to the T0 group. (**E**) Differential changes in signaling pathways within epithelial, T, and myeloid cells before and after UMSC treatment. The red rectangles highlight pathways associated with chemotaxis. Pairwise comparisons were performed between the T0 group and the other three groups. (**F**) Bubble plot illustrating the strength and significance of intercellular communication mediated by CXCL and CCL signaling pathways across the Ctrl, T2, and T6 groups. All the significant ligand-receptor pairs that contribute to CXCL and CCL signaling sending from DUOX2^+^CEACAM6^+^ enterocytes to T lymphocytes and myeloid cells were shown. The dot color and size represented the calculated communication probability and P-value, respectively. P-values are computed from one-sided permutation test.


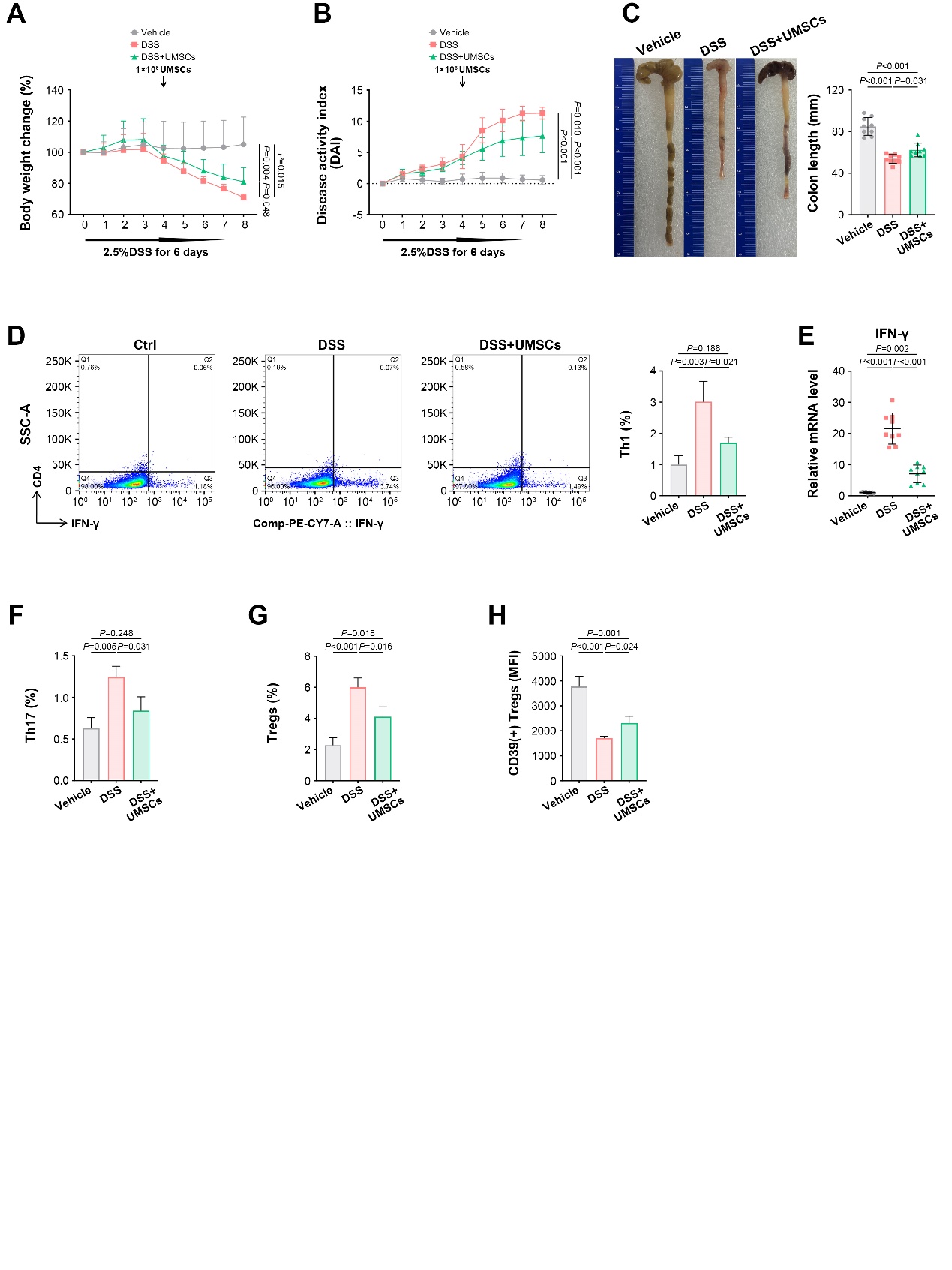


**Figure S6** **Changes of body weight, disease activity index, colon length, Th1, Th17, and Treg cells following UMSC treatment.** We employed DSS-induced colitis mouse models, administrated with UMSC treatment (Male mice, n=9 per group). (**A**) Body weight change; (**B**) disease activity index (DAI); and (**C**) representative images of colon in the three groups (vehicle, DSS, and DSS+UMSCs), with quantified analysis of colon length shown in the graph (right). (**D**) Representative images of flow cytometry for Th1 in spleen of the three groups. The percentage of Th1 in the graph is shown in the graph (right). (**E**) Statistics of mRNA level of IFN-γ in colon tissue of the three groups. (**F** and **G**) The percentage of Th17 cells and Tregs in the graph. (**H**) The mean fluorescence intensity (MFI) of CD39(+) Tregs in the three groups. Repeated ANOVA was used to compare the difference between the three groups in **A** and **B**. One-way ANOVA was used to compare the difference between the three groups in **C**, **E**, **F**, **G**, and **H**.

**References**

1 Zhang, Y., Lv, P., Li, Y. *et al.* Comparison of the biological characteristics of umbilical cord mesenchymal stem cells derived from the human heterosexual twins. *Differentiation*. 2020; 114: 1-12. doi:10.1016/j.diff.2020.05.005

2 Wu, M., Zhang, R., Zou, Q. *et al.* Comparison of the Biological Characteristics of Mesenchymal Stem Cells Derived from the Human Placenta and Umbilical Cord. *Sci Rep*. 2018; 8: 5014. doi:10.1038/s41598-018-23396-1

3 Wu, T., Hu, E., Xu, S. *et al.* clusterProfiler 4.0: A universal enrichment tool for interpreting omics data. *Innovation (Camb)*. 2021; 2: 100141. doi:10.1016/j.xinn.2021.100141

4 Trapnell, C., Cacchiarelli, D., Grimsby, J. *et al.* The dynamics and regulators of cell fate decisions are revealed by pseudotemporal ordering of single cells. *Nat Biotechnol*. 2014; 32: 381-386. doi:10.1038/nbt.2859

5 Jin, S., Guerrero-Juarez, C. F., Zhang, L. *et al.* Inference and analysis of cell-cell communication using CellChat. *Nature communications*. 2021; 12: 1088. doi:10.1038/s41467-021-21246-9

6 Van de Sande, B., Flerin, C., Davie, K. *et al.* A scalable SCENIC workflow for single-cell gene regulatory network analysis. *Nat Protoc*. 2020; 15: 2247-2276. doi:10.1038/s41596-020-0336-2
