# Supplementary material for: Reconstitution of T cell‐mediated immunity by umbilical cord‐derived mesenchymal stem cells in ulcerative colitis
Source: Clin Transl Med. 2025 Aug 21;15(8):e70452. doi: 10.1002/ctm2.70452 (PMC12371211; doi:10.1002/ctm2.70452)
Supplement: Supplementary file 2 — Supporting Information [file CTM2-15-e70452-s001.docx]

| **Supplementary Table 1. Baseline characteristics of active left-side UC patients** | |
| --- | --- |
| **Variables** | **Case (n=26)** |
| Sex [Male, n (%)] | 16 (61.5) |
| Age (in years, Mean±SD) | 49.5±12.2 |
| BMI [kg/m^2^, Mean±SD] | 23.1±4.3 |
| Duration of UC (years, Mean±SD) | 5.7±5.0 |
| Smoking [n (%)] |  |
| Never smokers | 19 (73.1) |
| Former smokers | 6 (23.1) |
| Current smokers | 1 (3.8) |
| Clinical severity [n (%)] |  |
| Moderate | 17 (65.4) |
| Severe | 9 (34.6) |
| History of therapy [n (%)] |  |
| 5-ASA alone | 13 (50.0) |
| 5-ASA + Glucocorticoids | 8 (30.8) |
| 5-ASA + Glucocorticoids + Immunosuppressant | 4 (15.4) |
| 5-ASA + Biological agents | 1 (3.8) |
